# Advancements in the utilization of immune checkpoint inhibitors for the treatment of gynecological tumors

**DOI:** 10.3389/fimmu.2026.1686568

**Published:** 2026-03-30

**Authors:** Zhonghua Su, Xuanyu Zhang, Jiayu Xu, Yue Shen

**Affiliations:** 1 Department of Gynecology, The First Hospital of China Medical University, Shenyang, Liaoning, China; 2 Department of Medical Record Management, Shengjing Hospital of China Medical University, Shenyang, Liaoning, China; 3 Department of Health Management, Shengjing Hospital of China Medical University, Shenyang, Liaoning, China; 4 Department of VIP In-Patient Ward, The First Hospital of China Medical University, Shenyang, Liaoning, China

**Keywords:** cervical cancer, endometrial cancer, immune checkpoint inhibitors, immunotherapy, ovarian cancer

## Abstract

Immunotherapy, as the fourth major cancer treatment method, has made breakthrough progress in the field of cancer therapy. Immune checkpoint inhibitors (ICIs) have been extensively utilized in the management of diverse malignant neoplasms, with a substantial corpus of clinical evidence and experience accumulating in the field of gynecological oncology. This article reviews the clinical application strategies of ICIs, either alone or in combination with radiotherapy and chemotherapy, targeted therapy, etc., for gynecological malignant tumors such as cervical cancer, endometrial cancer, ovarian cancer, and other malignancies. We summarized relevant progress from clinical trials with the aim of providing more effective precision immunotherapy strategies for patients with gynecological malignant tumors.

## 1 Introduction

Currently, immune checkpoint inhibitors (ICIs) are the most widely used and researched immunotherapy related to gynecological malignancies. By blocking the interaction between inhibitory receptors on T cells and their ligands, they relieve the immunosuppressive state in the tumor microenvironment and effector T-cell cytotoxic functions against tumors. This has become a revolutionary breakthrough in cancer treatment followity -50ng surgery, chemotherapy, radiotherapy, and targeted therapy. In the three major gynecological malignancies—endometrial cancer, cervical cancer, and ovarian cancer—the application of ICIs has made significant progress in the past 5 years, profoundly changing the landscape of clinical practice. This review aims to systematically examine the key research progress of ICIs in gynecological tumors, analyze the efficacy differences among various tumor types, explore the challenges and countermeasures faced in clinical applications, and discuss future development directions.

**Figure 1:**
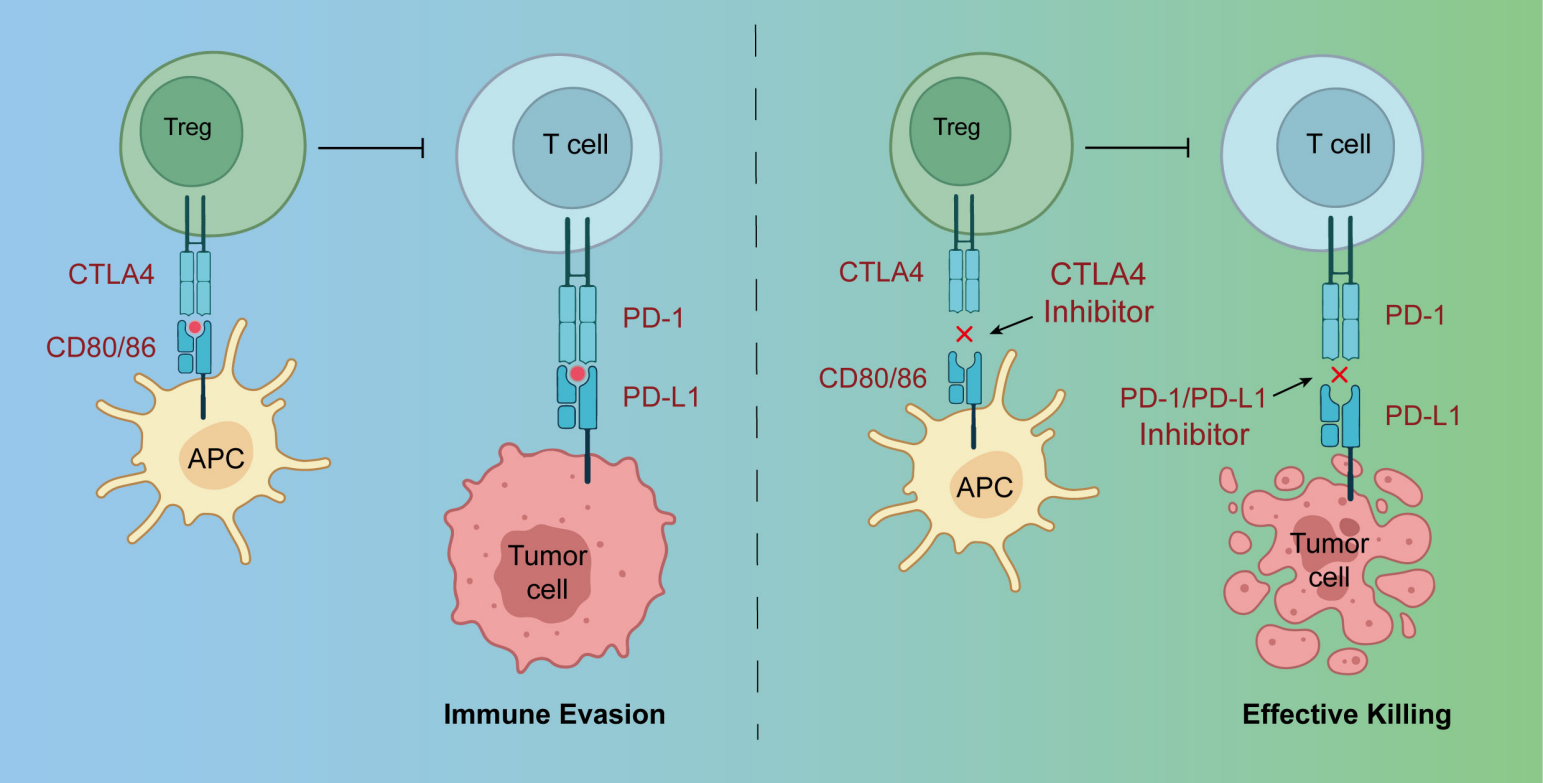
The mechanism of ICIs.

In 2006, Korman et al. (1) formally proposed the concept of immune checkpoints for the first time. Receptors on the surface of T cells recognize antigen signals and co-stimulatory molecules required for T-cell activation. Early-discovered co-stimulatory molecules, such as CD28, provide a second signal for T-cell activation, promoting T-cell activation, proliferation, and differentiation into effector T cells. Immune checkpoint molecules are expressed exclusively on activated T cells and serve as important brakes of immune responses. Upon binding their ligands, they inhibit the proliferation and differentiation of activated T cells. Multiple immune checkpoints have been identified, with programmed death-1 (PD-1), programmed death-ligand 1 (PD-L1), and cytotoxic T lymphocyte-associated antigen 4 (CTLA-4) receiving the most extensive and in-depth research (1, 2). We show the mechanism of ICIs in **Figure 1**.

### 1.1 CTLA-4 and its inhibitors

CTLA-4 is a transmembrane protein encoded by the *CTLA-4* gene, expressed on activated CD4+ T cells and CD8+ T cells, which binds to ligands CD80 (B7-1) and CD86 (B7-2) (3). CTLA-4 inhibits the activation of T-cell responses and mediates the suppressive function of regulatory T cells (Treg). In addition, CTLA-4 can mediate the binding of dendritic cells to CD80/CD86, inducing the expression of tryptophan-degrading enzyme indoleamine 2,3-dioxygenase, leading to TCR inhibition. CTLA-4 inhibitors reduce Treg suppression and enhance T-cell response activation. The CTLA-4 inhibitor ipilimumab was the first to be marketed, but its efficacy as a monotherapy is limited, and it has numerous adverse reactions. However, it shows certain promise when combined with PD-1 inhibitors, potentially enhancing the antitumor effects of the latter.

### 1.2 PD-1/PD-L1 and its inhibitors

PD-1 is a member of the CD28 superfamily with two ligands: PD-L1 (also known as CD274 or B7-H1) and PD-L2 (also known as CD273 or B7-DC) (4). Tumor cells express PD-L1, which binds to PD-1 and blocks T-cell activation and cytokine production. PD-1/PD-L1 inhibitors block this pathway by binding to PD-1/PD-L1, restoring immune cytotoxic functions.

### 1.3 PD-1/CTLA-4 bispecific antibodies or combination antibodies

Bispecific antibodies or combination antibodies targeting PD-1 and CTLA-4 can block the interactions between PD-1 and CTLA-4 with their ligands PD-L1/PD-L2 and B7-1/B7-2, thereby inhibiting the immunosuppressive responses of the PD-1 and CTLA-4 signaling pathways, promoting tumor-specific T-cell immune activation, and exerting antitumor effects (5).

## 2 Methods

This review intends to conduct a scoping review of the current research status of ICIs in gynecological tumors and address the following queries: 1) Currently, for which gynecological tumors are immune checkpoint inhibitors predominantly utilized? 2) What are the types of ICI drugs, medication protocols, and combination therapy strategies employed in clinical research? 3) What are the efficacy indicators and safety outcomes emphasized in the research reports? 4) How can patient groups that may benefit be efficiently identified? Are there any effective biomarkers available? 5) What are the current research gaps and future development directions in this domain?

We searched five electronic databases, including PubMed/MEDLINE, Cochrane Library, Embase, Web of Science, and China National Knowledge Infrastructure, from January 2015 to August 2025. The retrieval strategy adopts a combination of medical keywords and free words, including the combination of the following three types of keywords:

1. ICIs: “immune checkpoint inhibitor”, “PD-1 inhibitor”, “programmed cell death protein 1 inhibitor”, “PD-L1 inhibitor”, “programmed death-ligand 1 inhibitor”, “CTLA-4 inhibitor”, “cytotoxic T-lymphocyte-associated protein 4 inhibitor”, “Pembrolizumab”, “Keytruda”, “Nivolumab”, “Opdivo”, “Dostarlimab”, “Jemperli”, “Atezolizumab”, “Tecentriq”, “Durvalumab”, “Imfinzi”, “Cemiplimab”, “Libtayo”, “Ipilimumab”, “Yervoy”, “Cadonilimab”, “AK104”;
2. gynecological tumors: “gynecological cancer”, “gynecological tumor”, gynecological carcinoma”, “ovarian cancer”, “ovarian carcinoma”, “ovarian neoplasm”, “fallopian tube cancer”, “peritoneal cancer”, “endometrial cancer”, “endometrial carcinoma”, “endometrial neoplasm”, “uterine cancer”, “uterine carcinoma”, “cervical cancer”, “cervical carcinoma”, “cervical neoplasm”, “vulvar cancer”, “vulvar carcinoma”, “vulvar neoplasm”, “vaginal cancer”, “vaginal neoplasm”, “vaginal carcinoma;
3. research type: “clinical trial”, “phase II”, “phase III”, “observational study”, “cohort study”, “real-world study”, “retrospective study”, “prospective study”.

In addition, we manually searched the reference lists of relevant systematic reviews and meta-analyses, as well as conference abstracts from major academic conferences such as the American Society of Clinical Oncology, the European Society of Oncology, and the International Society of Gynecologic Oncology from 2020 to 2025 to obtain the latest unpublished research data.

## 3 Application in endometrial cancer

The expression ratio of PD-1/PD-L1 in endometrial cancer is relatively high, with the expression rate for endometrioid adenocarcinoma being 40%–80%, serous carcinoma 10%–68%, and clear cell carcinoma 23%–69% (6). At the same time, endometrial cancer is also a tumor with a high incidence of microsatellite instability-high (MSI-H) and/or deficient mismatch repair (dMMR), reaching 31%–37% (6), while tumor mutational burden (TMB-H) accounts for 11.2% (7). Advanced/recurrent endometrial cancer is a gynecological malignancy that benefits significantly from ICI treatment.

### 3.1 Monotherapy with ICIs

The application of ICIs alone in endometrial cancer mainly includes anti-PD-1 monoclonal antibodies: pembrolizumab, nivolumab, and dostarlimab, as well as anti-PD-L1 monoclonal antibodies atezolizumab and durvalumab, all of which have shown good efficacy in advanced or recurrent dMMR patients. The results of these trials are listed in **Table 1**. The specific outcomes of previously published trials are included in **Supplementary Table 1**.

**Table 1:** Clinical trials on immunotherapy for endometrial cancer.

Trial number	Treatments	Not yet recruiting	Recruiting	Active, not recruiting	Complete
NCT04273061	Atezolizumab (PD-L1)		✅		
NCT06278857	Dostarlimab (PD-1)		✅		
NCT02715284 (8)	Dostarlimab (PD-1)		✅		
NCT02630823	Pembrolizumab (PD-1)				✅
NCT01876511 (9)	Pembrolizumab (PD-1)				✅
NCT02054806 (10)	Pembrolizumab (PD-1)				✅
NCT02465060	Nivolumab (PD-1)			✅	
NCT01375842	Atezolizumab (PD-L1)			✅	
ACTRN12617000106336 (11)	Durvalumab (PD-L1)				✅
NCT02912572	Avelumab (PD-L1)			✅	
NCT02628067 (12)	Pembrolizumab (PD-1)			✅	

PD-1, programmed death-1; PD-L1, programmed death-ligand 1.

#### 3.1.1 Pembrolizumab

KEYNOTE-158 (NCT02628067) (12) is a non-randomized, open-label, phase II clinical trial in which cohorts D and K included patients with previously treated MSI-H/dMMR advanced/recurrent endometrial cancer receiving monotherapy with pembrolizumab. A total of 94 patients were recruited. Interim results indicated that with a median follow-up period of 54.5 months, the objective response rate (ORR) was 50%, the median progression-free survival (PFS) was 13.1 months, the median overall survival (OS) was 65.4 months, and the median duration of response (DOR) was 63.2 months. According to the results of the KEYNOTE-158 study (12), in 2022, the United States Food and Drug Administration (FDA) and European Medicines Agency (EMA) approved pembrolizumab for the treatment of patients with MSI-H/dMMR endometrial cancer who have disease progression following systemic therapy and are not candidates for curative surgery or radiation therapy.

#### 3.1.2 Nivolumab

The NCI-MATCH (EAY131) (NCT02465060) Z1D subgroup aims to evaluate nivolumab treatment in patients with dMMR non-colorectal tumors. This subgroup included 17 patients with endometrioid adenocarcinoma, endometrioid adenocarcinoma combined with other pathological types, and carcinosarcoma. Among them, interim results suggested that 13 were diagnosed with endometrioid adenocarcinoma, resulting in an ORR of 45.4%; out of the three patients who achieved complete response (CR), two had endometrioid adenocarcinoma (13). In studies on nivolumab and atezolizumab, PD-L1 expression was assessed, and subgroup analysis showed no correlation between treatment response rates and PD-L1 expression (11, 14). Presently, nivolumab is regarded as an alternative for second-line and subsequent therapies of recurrent/metastatic endometrial cancer (restricted to patients who have not undergone immunotherapy previously). However, its application in endometrial cancer has not been incorporated into the FDA’s approval.

#### 3.1.3 Dostarlimab

The GARNET (15) study is an open-label, single-arm, multi-cohort, phase I clinical trial (NCT02715284) investigating dostarlimab in patients with dMMR and proficient mismatch repair (pMMR) endometrial cancer. In this study, 104 patients with dMMR endometrial cancer who had received prior platinum-based chemotherapy were enrolled. Seventy-one patients with measurable lesions and more than 6 months of follow-up were included in the efficacy analysis. Interim results showed an ORR of 42.3%, with nine patients (12.7%) achieving a CR and 21 patients (29.6%) achieving a partial response (PR). The disease control rate (DCR) was 57.7%. The median PFS was 8.1 months, and the median OS was not reached. In the microsatellite stable (MSS)/pMMR cohort, which included 156 subjects, the ORR was 15.4%, the median PFS was 2.7 months, and the median OS was 16.9 months. In April 2021, the FDA granted accelerated approval to dostarlimab for the treatment of adult patients with dMMR recurrent or advanced endometrial cancer that has progressed on or following prior platinum-containing chemotherapy.

### 3.2 Combination therapy with ICIs

The efficacy of ICI monotherapy is poor in pMMR patients, and combination therapy is expected to improve its effectiveness. Combined therapy mainly includes combination chemotherapy, radiotherapy, targeted therapy, and other immunotherapies, aimed at leveraging the synergistic effects of different mechanisms to enhance efficacy. The results of these trials are listed in **Table 2**. The specific outcomes of previously published trials are included in **Supplementary Table 2**.

**Table 2:** Clinical trials on ICIs combined with other treatments for endometrial cancer.

Trial number	Treatments	Not yet recruiting	Recruiting	Active, not recruiting	Complete
NCT03192059 (16)	Pembrolizumab, radiation, and immunomodulatory cocktail				✅
NCT04652076	NP137 combined with pembrolizumab and/or chemotherapies			✅	
NCT04214067	Radiation ± pembrolizumab (PD-1)			✅	
NCT03914612 (17)	Pembrolizumab (PD-1), paclitaxel, and carboplatin			✅	
NCT06751277	QL1706 (PD-1/CTLA-4) combined with Chemotherapy	✅			
NCT05819892	Chemoradiation followed by chemotherapy + dostarlimab (PD-1)		✅		
NCT06532539	Cadonilimab (PD-1/CTLA-4) with chemoradiation		✅		
NCT03932409	Brachytherapy + pembrolizumab (PD-1) followed by 3 cycles of dose dense paclitaxel/q 21-day carboplatin + pembrolizumab (PD-1)			✅	
NCT03603184 (18)	Atezolizumab (PD-L1) and chemotherapy			✅	
NCT04634877 (19)	Pembrolizumab (PD-1) versus placebo in combination with adjuvant chemotherapy with or without radiotherapy			✅	
NCT06917092	QL1706 (PD-1/CTLA-4) combined with chemotherapy ± bevacizumab		✅		
NCT06253494	Pembrolizumab (PD-1), lenvatinib (TKI), and IL-15 superagonist N-803 in combination with HER2 targeting autologous dendritic cell (AdHER2DC) vaccine		✅		
NCT02501096 (20)	Lenvatinib (TKI) plus pembrolizumab (PD-1)				✅
NCT03517449 (21)	Lenvatinib (TKI) plus pembrolizumab (PD-1)				✅
NCT03903705	Fruquintinib (TKI) and sintilimab (PD-1)				✅
NCT04574284	TQB2450 (PD-L1) and anlotinib (TKI)				✅
NCT03884101 (22)	Lenvatinib (TKI) plus pembrolizumab (PD-1)				✅
ChiCTR2000031932 (23)	Camrelizumab (PD-1), rivoceranib (TKI)				✅
NCT02912572 (24)	Avelumab (PD-L1)/talazoparib (PARPi), or avelumab (PD-L1)/axitinib (TKI)			✅	
NCT03073525	Atezolizumab and Vigil				✅
NCT04065269	Ceralasertib (ATRi) + durvalumab (PD-L1)		✅		
NCT05572684	NC410 and pembrolizumab (PD-1)			✅	
NCT06989112	Trastuzumab deruxtecan (ADC) plus rilvegostomig (PD-1/TIGIT) or pembrolizumab (PD-1)		✅		
NCT04486352	Atezolizumab (PD-L1) + targeted agent		✅		
NCT06518564	Avelumab (PD-1) and M1774 (ATRi)		✅		
NCT03835819	Mirvetuximab soravtansine (FR-a) and pembrolizumab (PD-1)			✅	
NCT03629756	Etrumadenant (A2aR/A2bR antagonist-1) and zimberelimab (PD-1)				✅
NCT04269200 (25)	Durvalumab (PD-L1) ± olaparib (PARPi)			✅	
NCT03860272	Botensilimab (CTLA-4) and balstilimab (PD-1)			✅	

PD-1, programmed death-1; PD-L1, programmed death-ligand 1; CTLA-4, cytotoxic T lymphocyte-associated antigen 4; ICIs, immune checkpoint inhibitors; TKI, tyrosine kinase inhibitor; PARPi, poly(ADP-ribose) polymerase inhibitor.

#### 3.2.1 Combined anti-angiogenic therapy

Anti-angiogenic drugs can reverse the immunosuppressive effects induced by vascular endothelial growth factor, promoting the activation of T cells and other immune effector molecules; meanwhile, ICIs can enhance tumor vessel normalization by activating effector T cells, thereby increasing T-cell infiltration and cytotoxic function.

KEYNOTE-146/Study 111 (NCT02501096) final results showed that lenvatinib plus pembrolizumab increased efficacy in patients with advanced endometrial carcinoma who have experienced disease progression after prior systemic therapy, regardless of tumor MSI status (20). Based on this study, KEYNOTE-775/Study 309 (NCT03517449) (21) is a multicenter, randomized, controlled, phase III clinical trial comparing the efficacy and safety of pembrolizumab in combination with lenvatinib versus physician’s choice treatment (doxorubicin or weekly paclitaxel) for patients with advanced/recurrent endometrial carcinoma who have previously received at least one line of platinum-based therapy. A total of 827 patients were enrolled in the study, and the main endpoints were PFS and OS. The final results indicate that the median follow-up duration was 14.7 months. In the pembrolizumab combined with lenvatinib treatment group and the chemotherapy group, the median PFS for pMMR patients was 6.7 and 3.8 months, respectively (HR = 0.60, 95% CI: 0.50–0.72), while the median OS was 18.0 and 12.2 months, respectively (HR = 0.70, 95% CI: 0.58–0.83). The median DOR was reported as 9.3 and 5.7 months, with ORRs of 32.4% and 15.1%. The median PFS for the whole population was 7.3 and 3.8 months (HR = 0.56, 95% CI: 0.48–0.66), the median OS was 18.7 and 11.9 months (HR = 0.65, 95% CI: 0.55–0.77), the median DOR time was 12.9 and 5.7 months, with ORRs being 33.8% and 14.7%, respectively (21). The study indicates that in patients with advanced/recurrent endometrial cancer who have received at least one line of platinum-based therapy, the combination of pembrolizumab and lenvatinib can improve outcomes for all patients, including those with pMMR. Based on the study data, the U.S. FDA officially approved pembrolizumab in combination with lenvatinib in July 2021 for patients with advanced/recurrent non-MSI-H/dMMR endometrial carcinoma who have disease progression following systemic therapy and are not suitable for surgery or radiotherapy. In 2021, the EMA sanctioned the utilization of pembrolizumab in combination with lenvatinib for patients with advanced or recurrent endometrial cancer who have experienced disease progression following platinum-based chemotherapy and are ineligible for curative surgery or radiotherapy.

The phase III ENGOT-en9/LEAP-001 randomized controlled trial (NCT03884101) (22) aimed to evaluate the efficacy and safety of lenvatinib combined with pembrolizumab compared to paclitaxel combined with carboplatin as the first-line treatment for advanced or recurrent endometrial cancer, allowing the enrollment of patients who have experienced a recurrence more than 6 months after previous neoadjuvant/adjuvant chemotherapy. The final results indicate that in the overall population, there were no statistically significant differences in the primary endpoints of PFS and OS between the two groups. In pMMR patients, there were no significant differences in PFS and OS between the two groups. In dMMR patients, the ORRs for lenvatinib combined with pembrolizumab and chemotherapy were 72% and 58%, respectively, with median PFS of 31.8 and 9 months (HR = 0.61, 95% CI: 0.40–0.92), while the median OS was not reached for both groups (HR = 0.57, 95% CI: 0.36–0.91). For patients with a history of neoadjuvant or adjuvant chemotherapy, the PFS in the pMMR group (HR = 0.60, 95% CI: 0.37–0.97) and OS (HR = 0.67, 95% CI: 0.41–1.11), as well as PFS in the overall population (HR = 0.52, 95% CI: 0.33–0.82) and OS (HR = 0.64, 95% CI: 0.40–1.03), indicate that treatment with lenvatinib combined with pembrolizumab is more effective, further confirming that this combination can serve as an effective treatment option for patients who have progressed after previous chemotherapy (22).

In recent years, China has conducted several studies on the combination of ICIs and anti-angiogenic drugs for advanced/recurrent endometrial cancer patients who have previously received at least one line of platinum-based therapy. Multiple clinical trials have shown that ICIs combined with tyrosine kinase inhibitor (TKI) drugs achieve good efficacy in the treatment of platinum-based chemotherapy or intolerant pMMR advanced endometrial cancer patients. Common combinations include bemarituzumab combined with anlotinib (NCT04574284), sintilimab combined with fruquintinib (NCT03903705), and camrelizumab combined with apatinib (ChiCTR2000031932) (23), providing patients with new, safe, and effective treatment options.

#### 3.2.2 Combined chemotherapy

In recent years, an increasing number of clinical studies have suggested that ICIs combined with chemotherapy are expected to become the standard first-line treatment for patients with advanced metastatic or recurrent endometrial cancer. The synergistic mechanism of combined chemotherapy may be due to the ability of chemotherapy to enhance tumor antigen presentation and immunogenicity, induce PD-L1 expression in tumor cells, and improve the efficacy of chemotherapeutic agents by weakening baseline cell-mediated chemoresistance in effector T cells within the tumor microenvironment. Combined radiotherapy can promote the release and presentation of tumor antigens, enhancing immune recognition. At the same time, radiotherapy alters vascular permeability and chemokine secretion, recruiting effector immune cells such as T cells and NK cells into the core regions of tumors to facilitate immune cell infiltration (26).

KEYNOTE-868/NRG-GY018 (NCT03914612) (17) is a randomized, blinded, placebo-controlled, phase III clinical study that compares the efficacy and safety of pembrolizumab combined with standard chemotherapy versus placebo combined with chemotherapy (carboplatin and paclitaxel) in patients with advanced/recurrent first-line endometrial cancer. A total of 816 patients were enrolled in this study, with the primary endpoints being PFS for both dMMR and pMMR cohorts, having median follow-up times of 12 and 7.9 months, respectively. The interim results showed that in the pembrolizumab plus chemotherapy group and the placebo plus chemotherapy group, the median PFS for dMMR patients was not reached and 7.6 months, respectively (HR = 0.30, 95% CI: 0.19–0.48, p < 0.00001); for pMMR patients, the median PFS was 13.1 and 8.7 months (HR = 0.54, 95% CI: 0.41–0.71, p < 0.00001) (17). The OS data are still immature, with a median OS of 27.96 and 27.37 months for pMMR patients (HR = 0.79, 95% CI: 0.53–1.17, p = 0.1157), while the dMMR group has not yet reached median OS (HR = 0.55, 95% CI: 0.25–1.19, p = 0.0617) (17, 27). Although in the placebo group, 45% of pMMR patients and 54.5% of dMMR patients received subsequent treatment with anti-PD-1/PD-L1 inhibitors, there is still a trend toward benefit in OS for the pembrolizumab combined chemotherapy group. The study shows that patients can benefit significantly from the combination of pembrolizumab and chemotherapy, regardless of MMR status. In 2024, the FDA and EMA approved indications for pembrolizumab combined with standard chemotherapy (carboplatin and paclitaxel) for the treatment of advanced or recurrent endometrial cancer patients.

Phase III randomized controlled trial ENGOT-en7/MaNGO/AtTEnd (NCT03603184) (18) enrolled a total of 551 patients with advanced or recurrent disease who had not received prior systemic chemotherapy (including those previously treated with first-line platinum-based chemotherapy, provided that the interval since last platinum treatment was ≥6 months). The interim results compared the efficacy and safety of paclitaxel + carboplatin versus paclitaxel + carboplatin combined with atezolizumab, followed by maintenance therapy with atezolizumab. The results showed that in the dMMR population, the median PFS for the atezolizumab combination therapy group and the chemotherapy-only group were unavailable and 6.9 months (HR = 0.36, 95% CI: 0.23–0.57, p = 0.0005), respectively. In the overall population, the median PFS was 10.1 and 8.9 months, respectively (HR = 0.74, 95% CI: 0.61–0.91, p = 0.022). No significant benefit was observed in the pMMR type (HR = 0.92, 95% CI: 0.73–1.16). In the overall population, the median OS was 38.7 and 30.2 months, respectively (HR = 0.82, 95% CI: 0.63–1.07). In the dMMR population, the median OS was NR and 25.7 months (HR = 0.41, 95% CI: 0.22–0.76, p = 0.0026). No benefit was observed in the pMMR group (HR = 1.00, 95% CI: 0.74–1.35). The improvement in the overall population prognosis is primarily attributed to the clear benefit observed in the dMMR subgroup. However, its application in endometrial cancer has not been incorporated into the FDA’s approval.

The DUO-E/GOG-3041/ENGOT-EN10 (NCT04269200) (28) study is a phase III randomized controlled trial assessing the use of durvalumab in combination with carboplatin/paclitaxel chemotherapy, followed by first-line maintenance treatment with durvalumab ± olaparib in patients with newly diagnosed advanced or recurrent endometrial cancer. The study incorporated 718 newly diagnosed patients with stage III or IV, or recurrent endometrial cancer without prior systemic treatment (only allowing the inclusion of relapsed patients whose progression occurred ≥12 months after first-line adjuvant chemotherapy). Included patients were randomly assigned to group A (paclitaxel + carboplatin + placebo treatment, placebo maintenance therapy), group B (paclitaxel + carboplatin + durvalumab treatment, durvalumab maintenance therapy), and group C (paclitaxel + carboplatin + durvalumab treatment, durvalumab + olaparib maintenance therapy). The interim results showed that the median PFS for group A, group B, and group C was 9.6, 10.2, and 15.1 months, respectively; the HR for group C versus group B was 0.78 (95% CI: 0.61–0.99). In the pMMR population, the median PFS for group A, group B, and group C was 9.7, 9.9, and 15.0 months, respectively; the HR for group C vs. group B was 0.76 (95% CI: 0.59–0.99). In the overall population, compared to that in group A, OS in group C was significantly improved (not reached vs. 25.9 months, HR = 0.59, p = 0.003), while there was no statistically significant improvement in OS for group B (not reached vs. 25.9 months, HR = 0.77, p = 0.120). In dMMR patients, the addition of olaparib did not provide additional OS benefit compared to group B (HR = 0.84, 95% CI: 0.27–2.52); in pMMR patients, group C showed improved OS compared to group A (HR = 0.69, 95% CI: 0.47–1.00). Research hint: Durvalumab may improve prognosis in the dMMR subgroup; durvalumab + olaparib may enhance prognostic benefits in the pMMR subgroup (28). In 2024, the EMA granted approval for the combination therapy of durvalumab with carboplatin and paclitaxel, followed by the administration of durvalumab in conjunction with olaparib for patients with pMMR advanced or recurrent endometrial cancer. The FDA and the EMA have granted approval for the combination of durvalumab with carboplatin and paclitaxel, followed by durvalumab monotherapy, for patients with advanced or recurrent endometrial cancer presenting with dMMR.

The RUBY study (29) (NCT03981796) was a randomized, double-blind, vehicle-controlled, global, multicenter, phase III clinical trial designed to evaluate the efficacy and safety of dostarlimab in combination with chemotherapy (carboplatin + paclitaxel), followed by sequential dostarlimab monotherapy as maintenance treatment, in patients with primary advanced or recurrent endometrial cancer. A total of approximately 494 patients were enrolled in the study, including approximately 118 patients with MSI-H/dMMR subtypes. Results from the interim analysis of Part 1 demonstrated that in the MSI-H/dMMR population, the estimated 24-month PFS rate was 61.4% (95% CI: 46.3–73.4) in the dostarlimab group versus 15.7% (95% CI: 7.2–27.0) in the vehicle group (HR = 0.28, 95% CI: 0.16–0.50, p < 0.001). In the overall population, the 24-month PFS rate was 36.1% (95% CI: 29.3–42.9) in the dostarlimab group compared with 18.1% (95% CI: 13.0–23.9) in the vehicle group (HR = 0.64, 95% CI: 0.51–0.80, p < 0.001). The 24-month OS rate was 71.3% (95% CI: 64.5–77.1) in the dostarlimab group versus 56.0% (95% CI: 48.9–62.5) in the vehicle group (HR = 0.64, 95% CI: 0.46–0.87). These results demonstrate that dostarlimab combined with carboplatin–paclitaxel significantly improved PFS in patients with primary advanced or recurrent endometrial cancer, with pronounced benefit observed in the MSI-H/dMMR population. Based on these findings, the FDA approved dostarlimab in combination with chemotherapy for the treatment of adult patients with dMMR primary advanced or recurrent endometrial cancer in July 2023.

Adjuvant therapy for patients with a high risk of recurrence after uterine endometrial cancer surgery (such as those in stages III–IVA, or with specific pathological types or molecular characteristics) primarily consists of chemotherapy and radiotherapy; however, some patients still face the risk of recurrence, highlighting an urgent need for more effective treatment options. Immunotherapy combined with radiochemotherapy is expected to eliminate residual microscopic lesions after surgery and reduce the risk of recurrence.

The phase III ENGOT-en11/GOG-3053/KEYNOTE-B21 (19) randomized controlled trial assessed the efficacy of adjuvant chemotherapy (with or without radiotherapy) in combination with pembrolizumab among patients diagnosed with high-risk endometrial cancer. This study recruited 1,095 patients with stage I–IVA disease who exhibited no residual disease subsequent to surgery. The participants included high-risk stage I–II patients with non-endometrioid carcinoma or p53 abnormalities/TP53 mutations, among whom 281 cases presented with dMMR. The interim results showed that in the overall population, the combination therapy of pembrolizumab did not yield a statistically significant improvement in DFS (the 2-year DFS rate was 75% in the experimental group versus 76% in the control group; HR = 1.02, 95% CI: 0.79–1.32, p = 0.570). In the dMMR cohort, however, the combination therapy of pembrolizumab led to an improvement in DFS (the 2-year DFS rate was 92.4% in the experimental group versus 80.2% in the control group; HR = 0.31, 95% CI: 0.14–0.69). This study indicates that adjuvant chemotherapy (with or without radiotherapy) combined with pembrolizumab following surgery confers a DFS benefit for patients with dMMR.

In addition, the double antibody treatment regimen targeting PD-1 and CTLA-4 is still underway in clinical trials (NCT06751277, NCT06532539, and NCT06917092), with hopes of presenting promising prospects for patients with endometrial cancer.

Advanced or recurrent endometrial carcinoma, a gynecological malignancy, shows substantial benefits from ICI therapy. For patients with advanced or recurrent endometrial carcinoma who have experienced prior treatment failure and present with MSI-H/dMMR status, pembrolizumab is recommended, while nivolumab and durvalumab serve as alternative options. For patients with advanced or recurrent endometrial carcinoma who have failed prior therapy and exhibit MSS/pMMR status, the combination of pembrolizumab and lenvatinib is recommended. For recurrent endometrial carcinoma patients with MSS/pMMR status, the following treatment regimens are recommended: pembrolizumab plus lenvatinib, pembrolizumab plus anlotinib, sintilimab plus fruquintinib, or camrelizumab plus apatinib, and sintilimab plus anlotinib.

Regarding the first-line treatment of advanced or recurrent endometrial carcinoma, the combination of chemotherapy and pembrolizumab is recommended, along with concurrent pembrolizumab maintenance therapy. For the first-line treatment of advanced or recurrent endometrial carcinoma with MSI-H/dMMR, the following are recommended: the combination therapy of paclitaxel + carboplatin + durvalumab followed by durvalumab maintenance therapy; and the combination therapy of paclitaxel + carboplatin + atezolizumab followed by atezolizumab maintenance therapy. For the first-line treatment of patients with advanced or recurrent endometrial carcinoma with MSS/pMMR, the combination therapy of paclitaxel + carboplatin + durvalumab followed by durvalumab maintenance therapy is recommended. For adjuvant therapy of stage III–IV dMMR endometrial carcinoma, the combination therapy of paclitaxel + carboplatin + pembrolizumab and pembrolizumab maintenance therapy is recommended.

## 4 Application in cervical cancer

The proportion of MSI-H in cervical cancer patients is relatively low (2.62%), but the percentage of TMB-H is 14.9% (6). The expression rate of PD-L1 is high, ranging from 34.4% to 96.0% (30), suggesting that PD-1 inhibitors may be potential treatments for metastatic/recurrent cervical cancer.

### 4.1 Monotherapy with ICIs

ICI monotherapy is often used to explore treatments for metastatic/recurrent cervical cancer that has previously undergone at least first-line systemic therapy. The results of these trials are listed in **Table 3**. The specific outcomes of previously published trials are included in **Supplementary Table 3**.

**Table 3:** Clinical trials on immunotherapy for cervical cancer.

Trial number	Treatments	Not yet recruiting	Recruiting	Active, not recruiting	Complete
NCT04242199	INCB099280 (PD-L1)				✅
NCT05475171	Lorigerlimab (PD-1 + CTLA-4)		✅		
NCT04273061	Atezolizumab (PD-L1)		✅		
NCT02465060	Nivolumab (PD-1)			✅	
NCT02054806 (31)	Pembrolizumab (PD-1)				✅
NCT02628067 (32)	Pembrolizumab (PD-1)			✅	
NCT02257528 (29)	Nivolumab (PD-1)			✅	
NCT03676959 (33)	Socazolimab (PD-L1)				✅
NCT04590599	Sintilimab (PD-1)				✅
NCT03852251	AK104 (PD-1 + CTLA-4)				✅
NCT02488759 (34)	Nivolumab (PD-1)				✅

PD-1, programmed death-1; PD-L1, programmed death-ligand 1; CTLA-4, cytotoxic T lymphocyte-associated antigen 4.

#### 4.1.1 Pembrolizumab

The data from KEYNOTE series studies involving patients with metastatic/recurring cervical cancer show that the ORR of those who are PD-L1-positive receiving pembrolizumab treatment is 17.0% (31). The KEYNOTE-158 (NCT02628067) (12) and JapicCTI-163212 studies both conducted subgroup analyses based on the PD-L1 expression status of included cases, with results indicating that patients positive for PD-L1 expression have a better response to PD-1 inhibitors (35). The KEYNOTE-158 study was a single-arm, multi-cohort, multi-tumor, phase II clinical trial investigating pembrolizumab monotherapy. A total of 98 patients with cervical cancer who had received at least two prior lines of therapy were enrolled, among whom 82 patients (84%) had PD-L1-positive expression. Interim findings demonstrated that the ORRs in the entire population and the PD-L1-positive population were 14.3% and 17.1%, respectively. The median PFS was 2.1 months in both groups, while the median OS was 9.3 and 11.0 months, respectively (12). For patients with metastatic/relapsed cervical cancer, PD-L1 may be a more reliable biomarker. Based on the cervical cancer cohort from the KEYNOTE-158 (12) study, in June 2018, the U.S. FDA accelerated the approval of pembrolizumab for the treatment of patients with metastatic/recurrent cervical cancer who have received chemotherapy and whose disease has progressed during or after chemotherapy with a combined positive score (CPS) ≥ 1 for PD-L1 expression. In October 2021, the U.S. FDA granted full approval for this indication.

#### 4.1.2 Cemiplimab

Cemiplimab is a monoclonal antibody targeting PD-1. EMPOWER-Cervical 1/GOG-3016/ENGOT-cx9 (NCT03257267) (36) was a randomized, controlled, double-blind, phase III trial comparing cemiplimab with the investigator’s choice of chemotherapy in patients with recurrent or metastatic cervical cancer. A total of 608 patients were enrolled and randomized in a 1:1 ratio to the two treatment groups. The final outcomes demonstrated that the ORR was 16.4% for cemiplimab, in contrast to 6.3% for chemotherapy. The PFS was 2.8 months as compared to 2.9 months, and the median OS was 12.0 months in comparison with 8.5 months. Treatment-related adverse events (TRAEs) of grade 3 or higher occurred in 45.0% of patients in the cemiplimab group compared with 53.4% in the chemotherapy group. Long-term survival analysis demonstrated that cemiplimab significantly improved survival compared with the investigator’s choice of chemotherapy in patients with recurrent or metastatic cervical cancer. In 2022, cemiplimab was approved in Europe, Canada, Japan, and other regions for the treatment of recurrent or metastatic cervical cancer patients who have progressed during or after platinum-based chemotherapy.

#### 4.1.3 Nivolumab

Results from several phase I/II clinical studies (NCT02257528 (32) and NCT02488759 (34)) indicate that nivolumab has certain efficacy in the treatment of metastatic/recurrent cervical cancer, with an ORR ranging from 4.0% to 26.0% (14, 32).

Among these, the CheckMate 358 study (NCT02488759) is a phase I/II trial involving patients with recurrent/metastatic cervical cancer. Nineteen patients were subjected to nivolumab monotherapy at 240 mg every 2 weeks, 45 patients received nivolumab 3 mg/kg every 2 weeks plus ipilimumab 1 mg/kg every 6 weeks (NIVO3 + IPI1), and 112 patients received nivolumab 1 mg/kg plus ipilimumab 3 mg/kg every 3 weeks for four cycles, followed by nivolumab 240 mg every 2 weeks (NIVO1 + IPI3; 45 randomized and 67 in the expansion cohort). The final results showed that the median follow-up durations were 19.9, 12.6, and 16.7 months for the nivolumab monotherapy, NIVO3 + IPI1, and NIVO1 + IPI3 groups, respectively. The ORR were 26%, 31%, 40% (NIVO1 + IPI3 randomized cohort), and 38% (synthesis of randomized and expansion cohorts) (34). However, its application in endometrial cancer has not been incorporated into the FDA’s approval.

Additionally, multiple clinical trials have confirmed that monotherapies with capmatinib, socazolimab, and sintilimab provide significant benefits for patients with recurrent and metastatic cervical cancer (31, 37–39).

#### 4.1.4 PD-1/CTLA-4 bispecific antibodies or combination antibody therapy

AK104-201 (COMPASSION-03) (NCT03852251) was a multicenter, open-label, phase Ib/II clinical trial conducted in China. The cervical cancer cohort enrolled 111 patients with recurrent/metastatic cervical cancer who had previously failed platinum-containing chemotherapy. All patients were subjected to monotherapy with a PD-1/CTLA-4 bispecific antibody, and 99 were included in the efficacy analysis. The ORR was 32.3%. Specifically, the ORR was 42.9% in PD-L1-positive patients (CPS ≥ 1) and 16.7% in PD-L1-negative patients. The interim results indicated that among patients with prior bevacizumab treatment, the ORR was 28.0%, compared to 34.7% in those without prior bevacizumab exposure. The DCR was 51.5%. The median PFS was 3.7 months, and the median OS was not reached. The 18-month OS rate was 51.2% (40). This study demonstrated that patients could benefit from PD-1/CTLA-4 bispecific antibody treatment regardless of PD-L1 expression status or prior bevacizumab therapy. However, its application in cervical cancer has not been incorporated into the FDA’s approval.

The DUBHE-C-206 study is a single-arm, multicenter, phase II trial conducted in China, which enrolled 148 patients with recurrent or metastatic cervical cancer who had failed prior first-line platinum-based chemotherapy. These patients were subjected to treatment with a combination of PD-1 and CTLA-4 antibodies. The ORR was 33.8%, with rates of 37.1% in PD-L1-positive patients and 25.6% in PD-L1-negative patients. Among patients previously treated with bevacizumab, the ORR was 28.8%, compared to 37.1% in those without prior bevacizumab exposure. The DCR was 64.9%. The median PFS was 5.4 months, while the median OS was not reached (41). This study demonstrated that patients could benefit from this combination antibody therapy regardless of PD-L1 expression status or prior bevacizumab treatment. In 2024, China’s National Medical Products Administration (NMPA) approved iparomlimab and tuvonralimab for the treatment of recurrent or metastatic cervical cancer patients who have previously failed platinum-based chemotherapy.

In addition, the PD-1/CTLA-4 bispecific antibody Lorigerlimab (NCT05475171) is currently undergoing clinical trials, which may provide us with more treatment options.

### 4.2 Combination therapy with ICIs

Due to the unsatisfactory clinical benefits of ICI monotherapy for cervical cancer, several clinical studies have emerged that explore the combination of chemotherapy, radiotherapy, small-molecule TKIs, anti-angiogenic agents, and immunotherapy. These studies are intended to explore the combined treatment of ICIs in patients with previously treated progressive recurrent/metastatic cervical cancer, along with the outcomes of the first-line treatment for patients with metastatic/recurrent cervical cancer. The results of these trials are listed in **Table 4**. The specific outcomes of previously published trials are included in **Supplementary Table 4**. Compared to ICI monotherapy, the efficacy of ICI combination therapy has improved in previously treated metastatic/recurrent cervical cancer patients.

**Table 4:** Clinical trials on immunotherapy combined with other treatments for cervical cancer.

Trial number	Treatments	Not yet recruiting	Recruiting	Active, not recruiting	Complete
NCT04188860	Camrelizumab and albumin-bound paclitaxel				✅
NCT02635360	Pembrolizumab (PD-1), brachytherapy, and cisplatin				✅
NCT03192059 (16)	Pembrolizumab (PD-1), radiation, and immunomodulatory cocktail				✅
NCT06866951	Chemo-immunotherapy: paclitaxel (albumin-bound) and cisplatin and camrelizumab (PD-1)			✅	
NCT03614949	Stereotactic body radiation therapy and atezolizumab (PD-L1)			✅	
NCT04652076	NP137 combined with pembrolizumab (PD-1) and/or chemotherapies			✅	
NCT06727617	Serplulimab (PD-1) and chemoradiotherapy		✅		
NCT06416696	Toripalimab (PD-1) combined with chemoradiotherapy		✅		
NCT05492123	Nivolumab (PD-1)-ipilimumab (CTLA-4) followed by cisplatin-based chemoradiation		✅		
NCT07092696	Toripalimab (PD-1) and External Beam Radiation Therapy (EBRT)		✅		
NCT06093438	Toripalimab (PD-L1) and chemotherapy before definitive chemoradiation		✅		
NCT06986057	Iparomlimab and tuvonralimab (PD-1 + CTLA-4) combined with platinum-based chemotherapy	✅			
NCT03830866 (42)	Durvalumab (PD-L1) in combination with and following chemoradiotherapy compared to chemoradiotherapy alone				✅
NCT04221945 (43)	Chemoradiotherapy with or without pembrolizumab (PD-1)			✅	
NCT05588219	Tislelizumab (PD-1) combined with concurrent chemoradiotherapy		✅		
NCT03635567 (44)	Pembrolizumab (PD-1) plus chemotherapy				✅
NCT07003620	Neoadjuvant immunochemotherapy		✅		
NCT05173272	NACT and serplulimab (PD-1), chemoradiation, brachytherapy		✅		
NCT06916117	Neoadjuvant chemotherapy (paclitaxel and cisplatin) and sintilimab (PD-1), concurrent chemoradiotherapy, and consolidative immunotherapy	✅			
NCT0705539	Neoadjuvant chemo-immunotherapy: iparomlimab (PD-1/CD279/PDCD1) and tuvonralimab (CTLA-4) plus cisplatin, nab-paclitaxel for 1 cycle, and iparomlimab (PD-1) and tuvonralimab (CTLA-4) for 2 cycles	✅			
NCT06543576	1. Cisplatin, paclitaxel, pembrolizumab, bevacizumab (6 cycles); 2. EBRT, pembrolizumab, bevacizumab	✅			
NCT03989336	Manganese-primed sintilimab (PD-1) plus nab-paclitaxel, Platinum (nPP) chemotherapy				✅
NCT03556839 (45)	Platinum chemotherapy plus paclitaxel with bevacizumab and atezolizumab (PD-L1)			✅	
NCT05715840	SG001 (PD-1) plus chemotherapy ± bevacizumab	✅			
NCT04680988	SHR-1210 (PD-1) ± SHR-1020 (TKI) versus chemotherapy				✅
NCT07088731	Nab-paclitaxel, cisplatin, and sintilimab (PD-1)	✅			
NCT03816553 (46)	SHR-1210 (PD-1) in combination with apatinib (TKI)				✅
NCT03903705	Fruquintinib (TKI) monotherapy or plus sintilimab (PD-1)				✅
NCT02921269 (47)	Atezolizumab (PD-L1) and bevacizumab				✅
NCT03073525	Atezolizumab (PD-L1) and Vigil				✅
NCT04357873 (48)	Pembrolizumab (PD-1) and vorinostat				✅
NCT03946358 (49)	Atezolizumab (PD-L1) and UCPVax				✅
NCT04708470	PDS01ADC and bintrafusp alfa (PD-L1 and TGF-β)			✅	
NCT04432597 (50)	human papilloma virus (HPV) vaccine and M7824 (PD-L1 and TGF-β)			✅	
NCT05572684	NC410 and pembrolizumab (PD-1)			✅	
NCT06715241	Relatlimab (LAG-3) and nivolumab (PD-1)		✅		
NCT06238635	Dostarlimab (PD-1) and cobolimab (TIM-3)		✅		
NCT05944237	HTL0039732 (EP4) and atezolizumab (PD-L1)		✅		
NCT03228667	N-803 (IL-15) + pembrolizumab (PD-1)		✅		
NCT04977453	GI-101 (cytokine) and/or pembrolizumab (PD-1)/lenvatinib (TKI)/local radiotherapy		✅		
NCT06022029	ONM-501 (IFN) and cemiplimab (PD-1)		✅		
NCT06047379	NEO212 (TMZ + POH) and pembrolizumab (PD-1)		✅		
NCT04256213	Nivolumab (PD-1) and ipilimumab (CTLA-4)				✅
NCT04802876 (51)	Tislelizumab (PD-1) and spartalizumab (PD-1)			✅	
NCT06878222	Iparomlimab (PD-1/CD279/PDCD1) and tuvonralimab (CTLA-4)		✅		
NCT06095674	Balstilimab (PD-1) and botensilimab (CTLA-4)	✅			
NCT02488759 (52)	Nivolumab (PD-1) and ipilimumab (CTLA-4)				✅
NCT06511726	Cadonilimab (PD-1 + CTLA-4) and cisplatin and albumin-bound paclitaxel		✅		
NCT03755739	ICIs (PD-1 or PD-L1 and CTLA-4) and chemotherapeutic drug		✅		
NCT06289751	NACT and cadonilimab (PD-1 + CTLA-4)		✅		
NCT07035808	Iparomlimab and tuvonralimab (PD-1 + CTLA-4) and chemotherapy ± bevacizumab	✅			
NCT05235516	AK104/placebo (PD-1 + CTLA-4) combined with chemoradiotherapy			✅	
NCT06942416	Iparomlimab (PD-1) and tuvonralimab (CTLA-4), paclitaxel + cisplatin/carboplatin combined with radiotherapy		✅		
NCT04982237	AK104 (PD-1 + CTLA-4) plus platinum-containing chemotherapy ± bevacizumab			✅	
NCT03518606 (53)	Durvalumab (PD-L1), tremelimumab (CTLA-4), and metronomic vinorelbine (chemo)				✅
NCT05817214	Cadonilimab (PD-1 + CTLA-4), anlotinib (TKI), and granulocyte-macrophage colony-stimulating factor (GM-CSF)		✅		

PD-1, programmed death-1; PD-L1, programmed death-ligand 1; CTLA-4, cytotoxic T lymphocyte-associated antigen 4; NACT, neoadjuvant chemotherapy; TKI, tyrosine kinase inhibitor; ICIs, immune checkpoint inhibitors.

#### 4.2.1 Combined neoadjuvant therapy

Multiple phase II studies have explored neoadjuvant therapy combined with ICIs for patients with locally advanced cervical cancer (LACC), showing high disease response rates. Some patients may avoid adjuvant treatment based on postoperative pathology (54). The phase II NACI study investigated patients with PD-L1-positive (CPS ≥ 1) LACC (International Federation of Gynecology and Obstetrics (FIGO) 2018 IIB3, IIA2, and tumors ≥4 cm in diameter from stage IIC/IIIC) using neoadjuvant chemotherapy combined with camrelizumab. A total of 85 participants were enrolled; the interim results showed that the ORR for the full analysis set reached 98%, with a pathological complete response (pCR) rate of 38%. In the efficacy-evaluable population, ORR was 100%, and pCR was 39%. Among the surgical cohort, there were 81 operated patients and an additional group receiving postoperative adjuvant treatment comprising 20 individuals. At a median follow-up of 16 months, the 2-year event-free survival (EFS) rate was 94.6%, and the 2-year OS rate reached 97.9% (55). The integration of ICIs and chemotherapy for the neoadjuvant treatment of cervical cancer is intended to reduce tumor size, enhance the rates of surgical resection, and improve long-term prognosis. Meanwhile, it substantially decreases the necessity for postoperative radiotherapy in patients without raising new safety issues, thereby emerging as a promising novel avenue for future exploration. However, its application in cervical cancer has not been incorporated into the FDA’s approval.

#### 4.2.2 CCRT

Concurrent chemoradiotherapy (CCRT) is the standard treatment for LACC, but some patients still experience local or distant metastasis after treatment. Recently, studies have explored the use of ICIs in combination therapies for patients with LACC as first-line treatments alongside CCRT and even neoadjuvant chemotherapy. Examples include CCRT ± durvalumab (NCT03830866) (42), CCRT ± pembrolizumab (NCT04221945) (43), CCRT + toripalimab (NCT05588219), CCRT + cadonilimab (NCT05235516), and CCRT + tretinoin-based immunotherapies such as ChiCTR2000029068, all showing certain therapeutic effects.

The KEYNOTE-A18 study (NCT04221945) (43) is an international multicenter, randomized, double-blind, placebo-controlled, phase III clinical trial for high-risk LACC, aiming to evaluate the efficacy and safety of pembrolizumab combined with CCRT, with primary endpoints being PFS and OS. A total of 1,060 newly diagnosed high-risk LACC patients (FIGO 2014 stage I B2 to II B with positive lymph nodes or stage III to IVA) were included, among whom there were 299 Asian patients (149 from mainland China). The results of the first interim analysis showed that the 2-year PFS rates for the pembrolizumab combined with CCRT group and the CCRT group were 67.8% and 57.3% (HR = 0.70, 95% CI: 0.55–0.89, p = 0.0020), respectively, while the 2-year OS rates were 87.2% and 80.8% (HR = 0.73, 95% CI: 0.49–1.07). The final analysis results showed that the 3-year PFS rates were 64.3% and 55.6% (HR = 0.72, 95% CI: 0.59–0.87), and the 4-year OS rates were 75.4% and 70.2% (HR = 0.73, 95% CI: 0.57–0.94) (43). In the Chinese population, the pembrolizumab combined with CCRT group showed a superior improvement in PFS, with 2-year PFS rates of 86.3% and 58.5% (HR = 0.31, 95% CI: 0.15–0.64). In the FIGO 2014 stage III to IVA subgroup (a total of 601 cases worldwide, with 112 cases in mainland China), the 2-year PFS rates for the global population were 73% and 57%, respectively (HR = 0.57, 95% CI: 0.43–0.76, p < 0.0001). The study demonstrates that pembrolizumab combined with chemoradiotherapy can significantly improve PFS and OS in patients with high-risk LACC, especially those at FIGO 2014 stage III to IVA. In 2024, the FDA, EMA, and NMPA in China approved pembrolizumab for combination radiotherapy and chemotherapy in FIGO 2014 patients with stage A cervical cancer.

In radical radiotherapy, several phase I studies have explored immunotherapeutic modalities (e.g., GOTIC-018 study, NRG GY017 study, and NiCOL study), and the optimal approach of combining ICIs with radiotherapy remains to be further clarified.

#### 4.2.3 Combined anti-angiogenic drugs

The phase II study SHR-1210-II-217 (NCT04680988) is a randomized controlled trial comparing the combination of camrelizumab and fruquintinib (combination group) to camrelizumab monotherapy or investigator’s choice chemotherapy in patients with recurrent metastatic cervical cancer who had previously failed platinum-based chemotherapy. A total of 194 participants were enrolled, with a median follow-up time of 9.9 months. The interim findings indicated that the ORRs evaluated by the investigators for the camrelizumab plus fruquintinib group, the camrelizumab monotherapy group, and the investigator’s choice chemotherapy group were 42.9%, 22.2%, and 14.3%, respectively. The median PFS was reported as follows: 8.1, 4.1, and 2.9 months. The median follow-up was 13.6 months, and their median OS was 20.6, 14.9, and 13.9 months, respectively. A total of 105 cases were enrolled in the combined group, among which the ORR assessed by Blinded Independent Central Review (BICR) for 74 patients who had not previously received bevacizumab treatment was 44.6% (95% CI: 33.0–56.6), and the DCR was 81.1% (95% CI: 70.3–89.3). The median PFS was 6.4 months (95% CI: 6.2–12.4). In 2025, the NMPA of China approved the combination of camrelizumab and SHR-1020 for patients with recurrent or metastatic cervical cancer who had previously experienced treatment failure with platinum-based chemotherapy and had not received bevacizumab treatment.

In addition, there are treatment regimens that combine other anti-angiogenic drugs, which can achieve good efficacy in patients with advanced, recurrent, or metastatic cervical cancer. Examples include the following: SHR1210 combined with apatinib mesylate (NCT03816553) (56), tremelimumab combined with anlotinib (ChiCTR2400093200), and sintilimab combined with anlotinib (ChiCTR1900023015). These options provide more choices for clinical treatment.

#### 4.2.4 Combined chemotherapy and anti-angiogenesis drugs

The KEYNOTE-826 study (NCT03635567) (44) is a randomized, double-blind, placebo-controlled, phase III clinical trial exploring the efficacy and safety of pembrolizumab in combination with platinum-based chemotherapy (with or without bevacizumab) as the first-line treatment for patients with persistent, recurrent, or metastatic cervical cancer. The study enrolled 617 patients with persistent, recurrent, or metastatic cervical squamous cell carcinoma, adenocarcinoma, or adenosquamous carcinoma who had not previously received chemotherapy and were unsuitable for radiotherapy or surgery. Among these participants, 548 had CPS ≥ 1 and 317 had CPS ≥ 10; the primary endpoints were PFS and OS. The final results of the study suggested that among those with CPS ≥ 1, all enrolled patients, and those with CPS ≥ 10, the median PFS of patients in the pembrolizumab combined with platinum-containing chemotherapy group and the platinum-containing chemotherapy group were 10.4 and 8.2 months (HR = 0.62, 95% CI: 0.50–0.77, p < 0.001), 10.4 and 8.2 months (HR = 0.65, 95% CI: 0.53–0.79, p < 0.001), 10.4 months and 8.1 months, respectively (HR = 0.58, 95% CI: 0.44–0.77, p < 0.001). In the final OS analysis, for patients with CPS ≥ 1, all enrolled patients, and those with CPS ≥ 10, the median OS in the pembrolizumab plus platinum-based chemotherapy group compared to the platinum-based chemotherapy group was 28.6 vs. 16.5 months (HR = 0.60, 95% CI: 0.49–0.74), 26.4 vs. 16.8 months (HR = 0.63, 95% CI: 0.52–0.77), and 29.6 vs. 17.4 months (HR = 0.58, 95% CI: 0.44–0.78) (44). Subgroup analysis showed significant benefits in OS and PFS in the primary analysis population (PD-L1 CPS ≥ 1, CPS ≥ 10, and the entire population). The benefits of pembrolizumab were generally consistent across key subgroups, including histology, use of platinum-based therapy, use of bevacizumab, and prior radiochemotherapy alone. The study suggests that compared to platinum-based chemotherapy, pembrolizumab combined with platinum-based chemotherapy can significantly improve patients’ PFS and OS. In 2021, the FDA authorized pembrolizumab monotherapy, as well as the combination of pembrolizumab and platinum-based chemotherapy, with or without bevacizumab, as the first-line treatment for patients with persistent, recurrent, or metastatic cervical cancer whose tumors express PD-L1 (CPS ≥ 1).

The BEATcc study (NCT03556839) (45) is a phase III randomized controlled clinical trial for patients with metastatic, persistent, or recurrent cervical cancer, aimed at evaluating the efficacy and safety of atezolizumab in combination with platinum-based chemotherapy plus bevacizumab as the first-line treatment. A total of 410 cases were included in the study, with PFS and OS as the primary endpoints. The interim results showed that the median PFS for the atezolizumab combined with platinum-based chemotherapy plus bevacizumab group and the platinum-based chemotherapy plus bevacizumab group were 13.7 and 10.4 months, respectively (HR = 0.62, 95% CI: 0.49–0.78, p < 0.0001), while the median OS was 32.1 and 22.8 months, respectively (HR = 0.68, 95% CI: 0.52–0.88, p = 0.0046). This study suggests that the atezolizumab combination regimen significantly improves patients’ PFS and OS; however, all enrolled patients received concomitant bevacizumab without a placebo control or stratified analysis based on biomarker levels (PD-L1 expression). However, its application in cervical cancer has not been incorporated into the FDA’s approval.

KEYNOTE-826 demonstrated that adding pembrolizumab to chemotherapy ± bevacizumab significantly improves overall survival across the entire population. Building on this, BEATcc showed that the triple combination of chemotherapy + immunotherapy + anti-angiogenesis drugs outperforms the standard regimen of chemotherapy + bevacizumab.

#### 4.2.5 Dual antibody combination chemotherapy

The AK104–303 (57) (COMPASSION-16) study (NCT04982237) is a randomized, double-blind, placebo-controlled, phase III clinical trial designed to evaluate the efficacy and safety of cadonilimab combined with platinum-based chemotherapy (with or without bevacizumab) as the first-line treatment for persistent, recurrent, or metastatic cervical cancer. A total of 445 cases were enrolled in the study, including 116 with CPS < 1 and 312 with CPS ≥ 1. The primary study endpoints were PFS and OS. The interim research results show that among all enrolled patients, the median PFS for the cadonilimab combined with platinum-based chemotherapy group and the platinum-based chemotherapy group was 13.3 and 8.2 months (HR = 0.62, 95% CI: 0.49–0.79, p < 0.0001), respectively, while the median OS was not reached in one group and was 22.8 months in the other (HR = 0.64, 95% CI: 0.48–0.86, p = 0.0011). Regardless of whether bevacizumab was used or the PD-L1 expression status, all subgroups showed a benefit trend consistent with that of the overall population. Meanwhile, other key subgroups (age, histology, Eastern Cooperative Oncology Group (ECOG) status, history of CCRT, distant metastasis status, and type of platinum-based drugs) also showed a benefit trend consistent with the overall population. The study indicates that compared to platinum-based chemotherapy, the combination of cadonilimab and platinum-based chemotherapy can significantly improve PFS and OS in the overall patient population.

In 2025, China’s NMPA approved cadonilimab in combination with paclitaxel and platinum-based chemotherapy drugs, with or without bevacizumab, for the first-line treatment of persistent, recurrent, or metastatic cervical cancer.

Some clinical trial protocols for the first-line treatment of patients with persistent, recurrent, or metastatic cervical cancer also include a combination of apalutamide and toripalimab with chemotherapy ± bevacizumab (NCT05179317), SG001 combined with chemotherapy ± bevacizumab (NCT05715840), tislelizumab plus chemotherapy + bevacizumab (NCT05247619), and toripalimab in combination with chemotherapy + bevacizumab (ChiCTR2000029068 and NCT04973904). These studies confirm that immunotherapy combined with other treatment modalities can achieve good efficacy. Other second-line treatment options include Serplulimab combined with albumin paclitaxel (NCT04150575), tislelizumab plus albumin paclitaxel (NCT04341883), and cadonilimab combined with albumin paclitaxel (ChiCTR2300076740). Additionally, there are treatment regimens involving PD-1/PD-L1 inhibitors in combination with CTLA-4 inhibitors, such as nivolumab plus ipilimumab (NCT02488759), which provide more options for the second-line treatment of cervical cancer.

In addition, there are treatment regimens combining ICIs with antibody–drug conjugates (ADCs), such as cadonilimab + vedolizumab (ChiCTR2300076740), and second-generation PD-1 inhibitors—drugs that combine anti-PD-1 with anti-TGF-β—that are currently undergoing clinical trials. Let us look forward to new immunotherapy approaches making a significant impact in the treatment of cervical cancer.

ICIs are predominantly employed in patients with advanced, persistent, or recurrent cervical cancer, encompassing both monotherapy and combination therapy. These therapies augment efficacy via diverse antitumor mechanisms. PD-L1 expression continues to serve as a crucial immune-related biomarker for predicting the benefits of ICIs in cervical cancer patients. Moreover, for tumors with negative PD-L1 expression, immunohistochemistry can be utilized to detect MMR protein deficiency, while next-generation sequencing (NGS) can be employed to evaluate the TMB status. Multiple immune checkpoint inhibitors have been developed and incorporated into the clinical management of cervical cancer, with their indications expanding from the later-line to first-line treatments. Pembrolizumab remains one of the principal drugs recommended in clinical guidelines. Pembrolizumab is recommended for cervical cancer patients who exhibit high TMB, positive PD-L1 expression, or high MSI/negative dMMR and have received ≥2 prior lines of therapy. Pembrolizumab is also recommended for the ≥second-line treatment of recurrent/metastatic cervical cancer patients. Pembrolizumab in combination with platinum/paclitaxel ± bevacizumab is recommended as the first-line treatment for recurrent/metastatic cervical cancer patients with PD-L1 CPS ≥ 1, dMMR, or TMB-H. Pembrolizumab may be combined with concurrent chemoradiotherapy for newly diagnosed FIGO stage III–IVa cervical cancer patients.

## 5 Application in ovarian cancer

Most ovarian cancers are serous epithelial ovarian cancer, with MSI-H being rare, reported at only 1.37% (6), TMB-H at 1.47% (58), and PD-L1 expression in 10%–30% of cases (59). Ovarian cancer is generally considered to have the worst treatment efficacy in gynecological tumor immunotherapy.

### 5.1 Monotherapy with ICIs

Most clinical trials of ICI monotherapy for ovarian cancer are in phase I to II, and the ORR is low for patients with advanced/recurrent ovarian cancer, generally below 15% (60). The results of these trials are listed in **Table 5**. The specific outcomes of previously published trials are included in **Supplementary Table 5**.

**Table 5:** Clinical trials on immunotherapy for ovarian cancer.

Trial number	Treatments	Not yet recruiting	Recruiting	Active, not recruiting	Complete
NCT05788484	CDX-585 (PD-1 + ILT4)				✅
NCT04273061	Atezolizumab (PD-L1)		✅		
NCT02465060	Nivolumab (PD-1)			✅	
UMIN000005714	Pembrolizumab (PD-1)				
JapicCTI-153004 (61)	Nivolumab (PD-1)				
NCT02054806 (62)	Pembrolizumab (PD-1)				✅
EudraCT 2017-004168-36	Pembrolizumab (PD-1)				
NCT02674061 (63)	Pembrolizumab (PD-1)				
NCT02628067	Pembrolizumab (PD-1)				
NCT01375842 (64)	Atezolizumab (PD-L1)			✅	
NCT04047290	AK112 (PD-1/VEGF)				✅

PD-1, programmed death-1; PD-L1, programmed death-ligand 1.

In the KEYNOTE-158 study, the interim results showed that the ORR for patients with MSI-H/dMMR ovarian cancer was 33.3% (95% CI: 15.6%–55.3%), the median PFS was 2.2 weeks, and the median OS was 33.6 weeks. However, the study sample size was small, and the therapeutic efficacy was not satisfactory. The phase II PEACOCC study applied pembrolizumab treatment to patients with advanced gynecological clear cell carcinoma who had previously received ≥1 line of therapy, enrolling a total of 48 cases, including 41 cases of ovarian clear cell carcinoma. The PFS rate at 12 weeks was 43.8%, the ORR was 25.0%, the median PFS was 12.2 weeks, and the median OS was 71.0 weeks (65). However, its application in ovarian cancer has not been incorporated into the FDA’s approval.

Currently, two phase III clinical trials have yielded results. In the NINJA (66) trial targeting platinum-resistant recurrent patients, nivolumab demonstrated no statistically significant difference in OS compared to single-agent treatment with gemcitabine or liposomal doxorubicin. The JAVELIN Ovarian 200 (67) evaluated atezolizumab versus atezolizumab plus liposomal doxorubicin versus liposomal doxorubicin monotherapy in platinum-resistant recurrent patients. Neither PFS nor OS showed a statistically significant difference. The specific results of the main research are shown in **Supplementary Table 4**.

### 5.2 ICI combination therapy

The efficacy of ICI monotherapy for ovarian cancer is unsatisfactory. Some studies have explored the combination of ICIs with chemotherapy, and the results indicate that compared to the purely chemotherapy group, there was no benefit in PFS for patients receiving first-line or platinum-sensitive recurrent treatment and maintenance therapy with ICIs combined with chemotherapy (55, 68–73). For platinum-resistant recurrent ovarian cancer, some studies on combination chemotherapy have suggested that compared to that of previous monotherapy, the ORR of combined treatment has improved, but the DOR is very short. The results of these trials are listed in **Table 6**. The specific outcomes of previously published trials are included in **Supplementary Table 6**.

**Table 6:** Clinical trials on immunotherapy combined with other treatments for ovarian cancer.

Trial number	Treatments	Not yet recruiting	Recruiting	Active, not recruiting	Complete
NCT02608684	Pembrolizumab (PD-1), gemcitabine, and cisplatin				✅
NCT02431559 (71)	Motolimod, doxorubicin, and durvalumab (PD-L1)				✅
NCT02865811	Pembrolizumab (PD-1) combined with Pegylated Liposomal Doxorubicin (PLD)				✅
NCT03038100	Atezolizumab (PD-L1) versus placebo in combination with paclitaxel, carboplatin, and bevacizumab				✅
NCT03737643	Durvalumab (PD-L1) treatment in combination with chemotherapy and bevacizumab			✅	
NCT01633970	Atezolizumab (PD-L1) combination with bevacizumab and/or chemotherapy				✅
NCT02891824	Atezolizumab (PD-L1) vs. placebo with chemotherapy + bevacizumab				✅
NCT02853318 (72)	Pembrolizumab (PD-1), bevacizumab, and cyclophosphamide				✅
NCT05044871	Tislelizumab (PD-1) + bevacizumab + nab-paclitaxel				✅
NCT03734692	Rintatolimod, pembrolizumab, (PD-1) and cisplatin			✅	
NCT06600841	Adebrelimab (PD-L1) plus non-platinum chemotherapy and fuzuloparib (PARPi)	✅			
NCT05467670	Pembrolizumab (PD-1), ALX148, and doxorubicin		✅		
NCT03797326	Pembrolizumab (PD-1) plus lenvatinib (TKI)				✅
NCT05188781	Pembrolizumab (PD-1) and anlotinib (TKI)				✅
NCT04735861 (74)	Sintilimab (PD-1) plus bevacizumab				✅
NCT02873962 (75)	Nivolumab (PD-1)/bevacizumab/rucaparib (PARPi)			✅	
NCT03073525	Atezolizumab (PD-L1) and Vigil				✅
NCT02725489 (76)	Durvalumab (PD-L1) and Vigil				✅
NCT06108479	DF6215 (cytokine) and pembrolizumab (PD-1)		✅		
NCT04918186	Durvalumab (PD-L1) + BA3011; durvalumab + BA3021; ENB-003 + toripalimab (PD-1)	✅			
NCT04065269	Ceralasertib (ATRi) + durvalumab (PD-L1)		✅		
NCT05544929	KFA115 (immunomodulatory agent) + pembrolizumab (PD-1)		✅		
NCT04713514 (77)	OSE2101 ± pembrolizumab (PD-1)			✅	
NCT02571725	Olaparib (PARPi) and tremelimumab (CTLA-4)			✅	
NCT04034927	Olaparib (PARPi) and tremelimumab (CTLA-4)			✅	
NCT03836352	DPX-Survivac and pembrolizumab (PD-1)			✅	
NCT05572684	NC410 and pembrolizumab (PD-1)			✅	
NCT03602586	Pembrolizumab (PD-1) and epacadostat				✅
NCT03629756	Etrumadenant and zimberelimab (PD-1)				✅
NCT04580485	INCB106385 + INCMGA00012 (PD-1)				✅
NCT03861793	ALKS 4230 ± pembrolizumab (PD-1)				✅
NCT05544929	KFA115 (immunomodulatory agent) and pembrolizumab (PD-1)		✅		
NCT03761914	Galinpepimut-S (a multivalent WT1 analog peptide vaccine) and pembrolizumab (PD-1)				✅
NCT03740165	Pembrolizumab (PD-1) followed by maintenance with olaparib (PARPi)			✅	
NCT02734004 (78)	MEDI4736 (PD-L1) in combination with olaparib (PARPi)			✅	
NCT02657889 (79)	Niraparib (PARPi) in combination with pembrolizumab (PD-1)				✅
NCT04802876 (80)	Tislelizumab (CTLA-4) and spartalizumab (PD-1)			✅	
NCT03860272	Botensilimab (CTLA-4) and balstilimab (PD-1)			✅	
NCT02498600 (81)	Nivolumab (PD-1) with or without ipilimumab (CTLA-4)			✅	
NCT06940921	Low-Dose Radiation Therapy, SBRT, and cadonilimab (PD-1 + CTLA-4)			✅	
NCT03755739	ICIs (PD-1 or PD-L1 and CTLA-4) and chemotherapeutic drug		✅		
NCT03249142	Durvalumab (PD-L1) with or without tremelimumab (CTLA-4) and chemotherapy			✅	
NCT07002346	Iparomlimab and tuvonralimab (PD-1 + CTLA-4) combined with bevacizumab	✅			
NCT03267589	Durvalumab (PD-L1), tremelimumab (CTLA-4), MEDI 9447, MEDI 0562				✅

PD-1, programmed death-1; PD-L1, programmed death-ligand 1; CTLA-4, cytotoxic T lymphocyte-associated antigen 4; PARPi, poly(ADP-ribose) polymerase inhibitor; TKI, tyrosine kinase inhibitor; SBRT, stereotactic body radiation therapy; ICIs, immune checkpoint inhibitors.

The phase III KEYNOTE-B96 randomized controlled trial assessed the efficacy and safety of pembrolizumab combined with weekly paclitaxel (± bevacizumab) in comparison to placebo plus weekly paclitaxel (± bevacizumab) among patients with platinum-resistant recurrent ovarian cancer. PFS exhibited clinically significant improvements in both the intention-to-treat (ITT) population and the combined subgroup with CPS ≥ 1. Notably, the subgroup with CPS ≥ 1 achieved dual advantages in both PFS and OS. Specifically, at the first interim analysis, the PFS in the ITT cohort and the CPS ≥ 1 subgroup was 8.3 versus 6.4 months (HR = 0.70, 95% CI: 0.58–0.84, p < 0.0001) and 8.3 versus 7.2 months (HR = 0.72, 95% CI: 0.58–0.89, p = 0.0014), respectively. Regarding the HR, the ITT population demonstrated a 30% reduction in the risk of disease progression, while the CPS ≥ 1 subgroup showed a 28% reduction, which was consistent with the benefit observed in the overall population. These findings imply that subsequent biomarker stratification testing may not be necessary in clinical practice, thereby substantially enhancing treatment accessibility. At the second interim analysis, the median OS in the pembrolizumab group was significantly prolonged by approximately 4 months compared to the placebo group in the CPS ≥ 1 subgroup: 18.2 versus 14.0 months (HR = 0.76, 95% CI: 0.61–0.94, p = 0.0053).

The phase II BRIGHT study is a biomarker-based umbrella trial for platinum-resistant recurrent ovarian cancer, involving 108 patients in total. Group 2 consisted of patients with breast cancer susceptibility gene wild-type (BRCAwt) and CD8+ tumor-infiltrating lymphocytes (TILs) ≥3 (72 cases), who received treatment with tislelizumab plus bevacizumab and albumin-bound paclitaxel. The ORR was 48.6%, the median PFS was 7.3 months, and the median OS was 17.9 months.

Most of the studies on ICI combination targeted therapies are still ongoing, and a summary of the main studies with results can be found in **Supplementary Table 6**. The overall ORR of combination therapy with anti-angiogenic agents is 15% to 33%. The phase II INOVA study explored the use of sintilimab in combination with bevacizumab for the treatment of recurrent or persistent ovarian clear cell carcinoma (with at least one line of platinum-based chemotherapy, 61% being platinum-resistant). Among 37 evaluable patients, the ORR was 40.5%, with a median PFS of 6.9 months and a median OS of 28.2 months. Before joining the INOVA study, the final results showed that the self-controlled ORR of chemotherapy chosen by doctors was only 11.4%, with a median PFS of 3.2 months (74). In the MEDIOLA study, which evaluated the combination of olaparib and durvalumab in patients with gBRCA mutations sensitive to platinum, the final results showed that an ORR of 71.9% was achieved. However, in the TOPACIO/KEYNOTE-162 study involving niraparib combined with pembrolizumab in platinum-resistant patients, the final results showed that the ORR was only 18% (79).

In the NRG GY003 study, which evaluated combination therapy with other ICIs, the final results showed that patients receiving combined ipilimumab treatment had a significantly higher ORR compared to those treated with nivolumab monotherapy (31.4% vs. 12.2%) (OR = 3.28, 95% CI: 1.54–NE, p = 0.034). The median PFS was also significantly prolonged (3.9 vs. 2.0 months; HR = 0.528, 95% CI: 0.339–0.821, p = 0.004), and although there was an extension in median OS time as well, the difference did not reach statistical significance (28.1 vs. 21.8 months, HR = 0.789, 95% CI: 0.439–1.418, p = 0.43) (81). However, currently, the FDA has not approved the use of any of the above-mentioned ICIs for ovarian cancer.

First-line maintenance therapy with poly(ADP-ribose) polymerase (PARP) inhibitors significantly improves the prognosis of patients with advanced ovarian cancer harboring BRCA mutations. In recent years, some studies have explored the use of ICIs combined with PARP inhibitors (with or without bevacizumab) for first-line maintenance in BRCA wild-type patients, as detailed in **Supplementary Table 5**.

The DUO-O study results suggest that for patients with either BRCA wild-type homologous recombination deficiency (HRD)-positive or HRD-negative status, the combination of durvalumab with paclitaxel/carboplatin plus bevacizumab chemotherapy followed by maintenance treatment with durvalumab, olaparib, and bevacizumab can improve PFS compared to standard chemotherapy combined with bevacizumab followed by maintenance therapy with bevacizumab alone; however, no benefit in mid-term OS was observed. The first interim analysis results of the KEYLYNK-001 study show that pembrolizumab combined with chemotherapy followed by sequential treatment with pembrolizumab and olaparib (± bevacizumab) significantly improves PFS compared to chemotherapy alone (± bevacizumab), regardless of the patient’s PD-L1 expression status. This indicates that further exploration is needed for identifying populations that benefit from immunotherapy in ovarian cancer. *Post-hoc* exploratory analysis revealed that in the subgroup with CPS ≥ 10 and low loss of heterozygosity (LOH) without bevacizumab, single-agent pembrolizumab significantly improved PFS and OS.

The clinical trial results of the bispecific antibodies CDX-585 (PD-1 + ILT4) and AK112 (PD-1/VEGF) are yet to be published, which may bring new hope for ovarian cancer patients. In addition, a series of studies (AK104-IIT-003, AdORN, NEO-PEMBRO, NEO-PEMBROV, and OLAPem) are exploring the use of ICIs in combination with chemotherapy for neoadjuvant treatment of ovarian cancer and are still in phase I/II.

Based on the aforementioned studies, ICI monotherapy is not currently routinely advocated for patients with ovarian cancer. For patients with previously treated clear cell ovarian carcinoma, the combination of pembrolizumab or sintilimab with bevacizumab is recommended. For patients with platinum-resistant recurrent ovarian cancer, the combination of pembrolizumab with weekly paclitaxel (with or without bevacizumab) is recommended. For platinum-resistant recurrent patients with a BRCA wild-type (BRCAwt) status and CD8+ TILs ≥3, treatment with tislelizumab in combination with bevacizumab and albumin-bound paclitaxel is recommended.

## 6 Conclusion

ICIs have significantly revolutionized the treatment paradigm of gynecological cancers. Presently, the combination of ICIs with chemotherapy has emerged as the standard first-line therapeutic approach for advanced/recurrent cervical cancer and assumes a crucial role in specific subtypes of endometrial cancer.

Research findings suggest that patients who respond to ICIs predominantly display the following traits: PD-L1 expression, MSI-H, and TMB-H. These patients may undergo more extended remission phases. Essentially, this is because ICIs stimulate the body’s intrinsic immune system, establishing enduring immune memory that facilitates long-term monitoring and regulation of tumors.

The precise screening of beneficiary populations is of great significance. It not only maximizes the therapeutic efficacy and averts the risk of immune-related adverse reactions in non-responders but also optimizes the allocation of healthcare resources.

Future efforts focus on combination therapies to expand beneficiary populations. One of the most promising strategies involves integrating ICIs with ADCs. ADCs precisely deliver cytotoxic agents, inducing immunogenic cell death in tumor cells. Their synergistic effect with ICIs holds promise for converting “cold” tumors into “hot” tumors. Currently, multiple clinical trials combining ICIs with ADCs are underway for breast cancer, lung cancer, and urothelial carcinoma, indicating potential synergistic effects and clinical promise. This approach is anticipated to initiate a novel phase in the realm of gynecological oncology.

## 7 Limitations

First, this article predominantly relies on a retrospective summarization and analysis of published clinical trial outcomes. Its conclusions are contingent upon the quality and design of existing studies. The paper itself fails to offer novel prospective research data or conduct meta-analyses, thereby presenting limitations in the level of evidence.

Second, the discussion concerning treatment failure scenarios, the mechanisms of resistance to ICIs, and immune-related adverse events (irAEs) is relatively restricted. Moreover, although the paper identifies PD-L1 expression, MSI-H/dMMR, and TMB-H as biomarkers predictive of ICI efficacy, it does not adequately address the limitations of these markers.

In ovarian cancer, despite the presence of PD-L1-positive patients, the overall response rate to immunotherapy remains at a low level. In clinical practice, there are multiple PD-L1 detection antibodies (e.g., SP142, 28-8, 22C3, and SP263), which are associated with different drugs and platforms, and a unified standard for complete mutual recognition is lacking. The interpretation of PD-L1 involves diverse scoring systems (e.g., TPS and CPS), and the definition of the positive threshold also varies according to different clinical trials. This lack of standardization has resulted in substantial confusion in clinical decision-making, as the test results of the same patient may vary significantly across different platforms and interpretation standards.

Although the MSI-H/dMMR status can significantly predict the efficacy of immunotherapy, it has been noted in clinical practice that approximately one-third of MSI-H/dMMR patients still display primary resistance to PD-1 inhibitor monotherapy. In clinical testing, although the immunohistochemical detection of dMMR and the PCR/NGS detection of MSI-H show a high degree of consistency (research indicates approximately 91%), they are not entirely equivalent. The subtle differences in detection methods may influence our assessment of the degree of benefit to patients, and there is no consensus on how to interpret and make decisions for a small number of patients with inconsistent results.

There are significant disparities in the predictive value of TMB among different tumor types. TMB-H demonstrates good predictive efficacy in tumors with generally high TMB, such as melanoma and lung cancer. However, in gynecological tumors, the situation is far more complex. Particularly in ovarian cancer and triple-negative breast cancer, which have a low mutation rate, TMB-H cannot effectively predict the immune response. Unlike the “all-or-none” state of MSI-H, TMB is a continuous variable. What is the appropriate TMB value? Is the commonly used threshold of ≥10 mut/Mb applicable to all subtypes of gynecological tumors? This remains an unresolved issue. Setting the threshold too high may exclude some potentially beneficial patients; setting it too low may include patients in the ineffective population.

Finally, the article lacks head-to-head comparisons or systematic analyses of the relative advantages among these different combination regimens, which restricts its guidance for selecting optimal combination strategies for individual patients in clinical practice. In conclusion, this paper offers a valuable overview of the application of ICIs in gynecological cancers. Future research necessitates long-term data, in-depth exploration of resistance mechanisms and biomarkers, and direct comparisons of different combination therapies to further optimize treatment strategies.
