# Supplementary material for: Advancements in the utilization of immune checkpoint inhibitors for the treatment of gynecological tumors
Source: Front Immunol. 2026 Mar 30;17:1686568. doi: 10.3389/fimmu.2026.1686568 (PMC13071018; doi:10.3389/fimmu.2026.1686568)
Supplement: Supplementary file 1 [file Supplementaryfile1.zip › Supplementary Table 4.DOCX]

Supplementary Table 4. Clinical Trials results on ICIs Combined with Other Treatments for Cervical Cancer

| Title | Trial number | Treatments | Phase | group | Number(n) | ORR(95%CI) | DCR(95%CI) | mPFS(months, 95%CI) | mOS(months, 95%CI) |
| --- | --- | --- | --- | --- | --- | --- | --- | --- | --- |
| CALLA | NCT03830866 | Durvalumab(PD-L1) in Combination With and Following Chemoradiotherapy Compared to Chemoradiotherapy Alone | III | Durvalumab(PD-L1) in Combination With and Following Chemoradiotherapy vs Chemoradiotherapy Alone | 770(1:1) | - | NA | NR(HR=0.84, 95% CI: 0.65~1.08, P=0.17) | NR(HR=0.84,95% CI: 0.65~1.08, P=0.17) |
| KEYNOTE-A18 | NCT04221945 | Chemoradiotherapy With or Without Pembrolizumab(PD-1) | III | Chemoradiotherapy With Pembrolizumab vs Chemoradiotherapy | 1060 | 87.5 vs 83.7 | NA | 36months pfs%: 64.3% vs 55.6%（HR=0.72,95% CI:0.59~0.87） | 36months os%: 82.6% vs 74.8%, HR=0.67(95% CI: 0.50~0.90) |
| KEYNOTE-826 | NCT03635567 | Pembrolizumab(PD-1) Plus Chemotherapy | III | Chemotherapy and Pembrolizumab vs Chemotherapy | 617(1∶1) | 66.2 vs 51.5 | NA | 10.4 vs 8.2(HR=0.61, 95%CI:0.50~0.74, P＜0.0001) | 24months os%: 52.1%vs 38.7%(HR=0.63, 95%CI: 0.52~0.77, P＜0.0001) |
| BEATcc | NCT03556839 | Platinum Chemotherapy Plus Paclitaxel With Bevacizumab and Atezolizumab(PD-L1) | III | Chemotherapy and Atezolizumab vs Chemotherapy | 410(1∶1) | 84 vs 72 | - | 13.7 vs 10.4 (HR=0.62,95%CI: 0.49~0.78, P＜0.0001) | 32.1 vs 22.8(HR=0.68，95%CI: 0.52~0.88, P=0.0046) |
| CLAP | NCT03816553 | SHR-1210(PD-1) in Combination With Apatinib(TKI) | II | single-arm | 45 | 55.6(40.0~70.4) | 82.2(70.6～93.8) | 8.9(5.6~18.1) | 20.3(9.3~36.9) |
| - | NCT02921269 | Atezolizumab(PD-L1) and Bevacizumab | II | single-arm | 10 | NA | 60 | 2.9(1.8~6.0) | 8.9(3.4~21.9) |
| PEVOsq | NCT04357873 | Pembrolizumab(PD-1) and vorinostat | II | single-arm | 26 | 39(20~62) | - | 4.2 | 10.3 |
| CHECKMATE358 | NCT02488759 | Nivolumab(PD-1) and Ipilimumab(CTLA-4) | I/II | single-arm | 19 | 26(9~51) | - | 5.1(1.9~9.1) | 21.6(8.3~46.9) |
| - | NCT03518606 | Durvalumab(PD-L1) and Tremelimumab(CTLA-4) and metronomic Vinorelbine(chemo) | I/II | single-arm | 31 | 41.9 | - | 7.4(1.9~23) | 13.1(8.5～NE) |
| PRIMMO | NCT03192059 | Pembrolizumab(PD-1) and Radiation and Immune Modulatory Cocktail | II | single-arm | 18 | 11.1 | - | 1.03(1.03~6.4) | 9.9(3.75~16.75) |
