# Supplementary material for: Advancements in the utilization of immune checkpoint inhibitors for the treatment of gynecological tumors
Source: Front Immunol. 2026 Mar 30;17:1686568. doi: 10.3389/fimmu.2026.1686568 (PMC13071018; doi:10.3389/fimmu.2026.1686568)
Supplement: Supplementary file 1 [file Supplementaryfile1.zip › Supplementary Table 5.DOCX]

Supplementary Table 5. Clinical Trials on Immunotherapy for Ovarian Cancer

| Title | Trial number | Treatments | Phase | group | Number(n) | ORR(95%CI) | DCR(95%CI) | mPFS(months, 95%CI) | mOS(months, 95%CI) |
| --- | --- | --- | --- | --- | --- | --- | --- | --- | --- |
| NINJA | JapicCTI-153004 | Nivolumab (PD-1) | III | Nivolumab vs chemotherapy | 316(1:1) | 7.6(3.5~13.9) vs 13.2(7.6~20.8) | 36.1 vs 60.5 | 2.0(1.9~2.2) vs 3.8(3.6~4.2) HR=1.5，P=0.002 | 10.1(8.3~14.1) vs 12.1(9.3~15.3), HR=1.0, P=0.808 |
| - | UMIN000005714 | Nivolumab (PD-1) | II | single-arm | 20 | 15(3.2~37.9) | 45(23.1~68.5) | 3.5(1.7~3.9) | 20(7.0~NR) |
| KEYNOTE-028 | NCT02054806 | Pembrolizumab(PD-1) | IB | single-arm | 26 | 11.5(2.4~30.2) | 38.4 | 1.9(1.8~3.5) | 13.8(6.7~18.8) |
| KEYNOTE-100 | NCT02674061 | Pembrolizumab(PD-1) | II | single-arm | 376 | 8.5(5.9～11.8) | 22.1(18.0~26.6) | cohort A：2.1(2.1~2.2) cohort B：2.1(2.1~2.6) | cohort A：18.7(17.0~22.5) cohort B：17.6(3.3~24.4) |
| KEYNOTE-158 | NCT02628067 | Pembrolizumab(PD-1) | II | single-arm | 15 | 33.3(11.8~61.6) | NR | 2.3(1.9~6.2) | NR(3.8~NR) |
| PEACOCC | EudraCT 2017-004168-36 | Pembrolizumab(PD-1） | II | single-arm | 48 | 25.0(14～40) | 58 | 2.7(1.3～5.4) | 14.8(6.7～28.2) |
| - | NCT01375842 | Atezolizumab(PD-L1) | IA | single-arm | 10 | 22.2(2.8~60.0) | 22.2 | 2.9(1.3~5.5) | 11.3(5.5~27.7) |
