# Supplementary material for: Advancements in the utilization of immune checkpoint inhibitors for the treatment of gynecological tumors
Source: Front Immunol. 2026 Mar 30;17:1686568. doi: 10.3389/fimmu.2026.1686568 (PMC13071018; doi:10.3389/fimmu.2026.1686568)
Supplement: Supplementary file 1 [file Supplementaryfile1.zip › Supplementary Table 6.DOCX]

Supplementary Table 6. Clinical Trials results on ICIs Combined with Other Treatments for Ovarian Cancer

| Title | Trial number | Treatments | Phase | group | Number(n) | ORR(95%CI) | DCR(95%CI) | mPFS(months, 95%CI) | mOS(months, 95%CI) |
| --- | --- | --- | --- | --- | --- | --- | --- | --- | --- |
| - | NCT02865811 | Pembrolizumab(PD-1) Combined With PLD | II | single-arm | 23 | 26.1(10.2~48.4) | CBR:52.2(30.6~73.2) | 8.1(1.7~14.7) | 18.3(9.4~31.5) |
| IMagyn050/GOG3015/EN GOT-OV39 | NCT03038100 | Atezolizumab(PD-L1) Versus Placebo in Combination With Paclitaxel, Carboplatin, and Bevacizumab | III | Atezolizumab(PD-L1) vs Placebo With Paclitaxel, Carboplatin, and Bevacizumab | 1301(1:1) | 93(89~96) vs 89(84~92) | NA | 19.5(18.1~20.8) vs 18.4(17.2~19.8), HR=0.92(0.79~1.07)，P=0.2785 | NE |
| - | NCT01633970 | Atezolizumab(PD-L1) Combination With Bevacizumab and/​or With Chemotherapy | IB | single-arm | 20 | 15(3.2~37.9) | 55(31.5~76.9) | 4.9(1.2~20.2) | 10.2(1.2~26.6) |
| ATALANTE | NCT02891824 | Atezolizumab(PD-L1) vs Placebo With Chemotherapy+Bevacizumab | III | Atezolizumab(PD-L1) vs Placebo With Chemotherapy and Bevacizumab | 614(2:1) | NA | NA | 13.5(12.2~14.2) vs 11.3(11.0~13.5), P=0.041, HR=0.83(0.69~0.99) | 35.5(32.4~41.3) vs 30.6(27.9~33.6) |
| - | NCT02853318 | Pembrolizumab(PD-1), Bevacizumab, and Cyclophosphamide | II | single-arm | 40 | 47.5(90%CI:34.9~60.3) | 95 | 10.0(90%CI:6.5~17.4) | NA |
| BRIGHT | NCT05044871 | Tislelizumab(PD1)+ Bevacizumab + Nab-paclitaxel | II | Tislelizumab(PD1)+Bevacizumab + Nab-paclitaxel vs Bevacizumab + Nab-paclitaxel | 72 | 48.6(36.4~60.8) | 78.6(67.1~87.5) | 7.3(5.0~9.2) | 17.9(12.6~NA) |
| LEAP-005 | NCT03797326 | Pembrolizumab(PD-1) Plus Lenvatinib(TKI) | II | single-arm | 31 | 26(12~45) | 77 | 6.2(4.0~8.5) | 21.3(11.7~32.3) |
| INOVA | NCT04735861 | Sintilimab(PD-1) Plus Bevacizumab | II | single-arm | 37 | 40.5(24.8~57.9 ) | 73.0(55.9~86.2) | 6.9(5.3~8.1) | 28.2(23.8~NR) |
| - | NCT02873962 | Nivolumab(PD-1)/​Bevacizumab/​Rucaparib(PARPi) | II | single-arm | 38 | 28.9(15.4~45.9) | 55.3 | 9.4(6.7~NA) | NA |
| - | NCT02725489 | Durvalumab(PD-L1) and vigil | I/II | single-arm | 5 | - | - | NR | NR |
| MEDIOLA | NCT02734004 | MEDI4736(PD-L1) in Combination With Olaparib(PARPi) | II | single-arm | 32 | 71.9(53.25~86.25) | 65.6 (90%CI:49.6~79.4) | 11.1(8.2~15.9) | NR |
| TOPACIO/KEYNOTE-162 | NCT02657889 | Niraparib(PARPi) in Combination With Pembrolizumab(PD-1) | I/II | single-arm | 60 | 18(90%CI:11~29) | 65(90%CI:54～75) | 3.4(2.1~5.1) | NA |
| NRG GY003 | NCT02498600 | Nivolumab(PD-1) With or Without Ipilimumab(CTLA-4) | II | Nivolumab vs Nivolumab and Ipilimumab | 100(1:1) | 12.2 vs 31.4 | NA | 2.0 vs 3.9(HR=0.528, 0.339~0.821, P=0.004) | 21.8 vs 28.1(HR=0.789,0.439~1.418,P=0.43) |
