# Supplementary material for: Advancements in the utilization of immune checkpoint inhibitors for the treatment of gynecological tumors
Source: Front Immunol. 2026 Mar 30;17:1686568. doi: 10.3389/fimmu.2026.1686568 (PMC13071018; doi:10.3389/fimmu.2026.1686568)
Supplement: Supplementary file 1 [file Supplementaryfile1.zip › Supplementary Table 3.DOCX]

Supplementary Table 3. Clinical Trials results on ICIs for Cervical Cancer

| Title | Trial number | Treatments | Phase | group | Number(n) | ORR(95%CI) | DCR(95%CI) | mPFS(months, 95%CI) | mOS(months, 95%CI) |
| --- | --- | --- | --- | --- | --- | --- | --- | --- | --- |
| KEYNOTE-028 | NCT02054806 | Pembrolizumab(PD-1) | IB | single-arm | 24 | 17(5~37) | 30 | 2(2~3) | 11(4~15) |
| KEYNOTE-158 | NCT02628067 | Pembrolizumab(PD-1） | II | single-arm | 99 | 14.3(8~22.8) | 30.6 | 2.1(2.1~2.2) | 9.3(7.6~11.7) |
| NRG-GY002 | NCT02257528 | Nivolumab(PD-1) | II | single-arm | 25 | 4(0.4~22.9) | 40 | 3.5(1.9~5.1) | 14.5(8.3~26.8) |
| - | NCT03676959 | Socazolimab (PD-L1） | I | single-arm | 103 | 19.8 | 49.5 | 4.44 | 15.84 |
