# Supplementary material for: Advancements in the utilization of immune checkpoint inhibitors for the treatment of gynecological tumors
Source: Front Immunol. 2026 Mar 30;17:1686568. doi: 10.3389/fimmu.2026.1686568 (PMC13071018; doi:10.3389/fimmu.2026.1686568)
Supplement: Supplementary file 1 [file Supplementaryfile1.zip › Supplementary Table 2.DOCX]

Supplementary Table 2. Clinical Trials results on ICIs Combined with Other Treatments for Endometrial Cancer

| Title | Trial number | Treatments | Phase | group | Number(n) | ORR(95%CI) | DCR(95%CI) | mPFS(months, 95%CI) | mOS(months, 95%CI) |
| --- | --- | --- | --- | --- | --- | --- | --- | --- | --- |
| PRIMMO | NCT03192059 | Pembrolizumab and Radiation and Immune Modulatory Cocktail | II | single-arm | 25 | 12 | - | 0.9(0.9~3.85) | 9.35(4.75~50.3) |
| NRG-GY018/KEYNOTE-868 | NCT03914612 | Pembrolizumab(PD-1) ,Paclitaxel and Carboplatin | III | Pembrolizumab combined with Paclitaxel and Carboplatin vs chemotherapy(Paclitaxel or Carboplatin) (1:1) | 816 | pMMR:71 vs 58 dMMR:82 vs 71 | - | dMMR:NR vs 7.6,HR=0.30,95%CI:0.19~0.48,P<0.00001 pMMR:13.1 vs 8.7,HR=0.54,95%CI:0.41~0.71,P<0.00001 ITT:18.8 vs 8.5 | dMMR:NR,HR=0.55,95%CI: 0.25~1.19,P=0.0617; pMMR: 27.96 vs 27.37, HR=0.79,95%CI: 0.53~1.17, P=0.1157 |
| AtTEnd | NCT03603184 | Atezolizumab(PD-L1) and chemotherapy | III | Atezolizumab and chemotherapy vs chemotherapy(1:1) | 551 | ITT:75 vs 74.6 dMMR:82.4 vs 75.7 | - | ITT:10.1(95%CI:9.5~12.3 vs 8.9,HR=0.74,95%CI: 0.61~0.91, P=0.022 dMMR:NE vs 6.9, HR=0.36, 95% CI:0.23~0.57,P=0.0005 | ITT:38.7(95% CI:30.6~NE) vs 30.2 (25.0~37.2), HR=0.82, 95%CI: 0.63~1.07 dMMR: NE vs 25.7,HR=0.41, 95%CI: 0.22~0.76, P=0.0026 |
| ENGOT-en11/GOG- 3053/KEYNOTE-B21 | NCT04634877 | Pembrolizumab(PD-1)Versus Placebo in Combination With Adjuvant Chemotherapy With or Without Radiotherapy | III | Pembrolizumab combined with Paclitaxel and Carboplatin and/or Radiotherapy vs Paclitaxel and Carboplatin and/or Radiotherapy | 1095 | - | - | ITT  DFS:HR=1.02, 95%CI:0.79~1.32, P=0.57 | NR |
| KEYNOTE-146 | NCT02501096 | Lenvatinib (TKI) Plus Pembrolizumab(PD-1) | II | single-arm | 108 | 39.8(30.5~49.7) MSI-H/dMMR:63.6(30.8~89.1) MSS/pMMR:38.3(28.5~48.9) | ITT: 82.4(73.9~89.1) MSI-H/dMMR: 90.9(58.7~99.8) MSS/pMMR: 81.9(72.6~89.1) | ITT:7.4(5.2～8.7) MSI-H/dMMR: 26.4(4.0～NE) MSS/pMMR: 7.4(4.4～7.6) | ITT:17.7(15.5~25.8) MSI-H/dMMR:NE(7.4~NE) MSS/pMMR:17.2(15.0~25.8) |
| KEYNOTE-775 | NCT03517449 | Lenvatinib (TKI) Plus Pembrolizumab(PD-1) | III | Lenvatinib and Pembrolizumab vs Doxorubicin/Paclitaxel | 827 | ITT: 33.8 vs 14.7 pMMR: 32.4 vs 15.1 dMMR:41.5 vs 12.3 | ITT:72.3 vs 46.6 pMMR:72.0 vs 46.4 dMMR: 73.8 vs 47.7 | ITT:7.3 vs 3.8,HR=0.56,95%CI:0.48~0.66; pMMR:6.7 vs 3.8,HR =0.60,95% CI:0.50~ 0.72; dMMR:10.7 vs 3.7,HR=0.39: 95% CI:0.25~0.60; | ITT:18.7 vs 11.9, HR=0.65; 95% CI: 0.55～0.77; pMMR: 18.0 vs 12.2,HR=0.70,95% CI: 0.58~0.83; dMMR:31.9 vs 8.6, HR=0.43, 95% CI: 0.28~0.68; |
| LEAP-001 | NCT03884101 | Lenvatinib (TKI) Plus Pembrolizumab(PD-1) | III | Lenvatinib and Pembrolizumab vs chemotherapy(Paclitaxel or Carboplatin） | 842 | ITT: 56 vs 55 pMMR: 51 vs55 dMMR:72 vs58 | ITT:84 vs 84 pMMR:83 vs 85 dMMR:87 vs 81 | ITT:12.5 vs 10.2,HR=0.91; 95% CI: 0.76~ 1.09 pMMR:9.6 vs 10.2,HR=0.99;95% CI:0.82~ 1.21 dMMR:31.8 vs 9.0,HR=0.61;95% CI: 0.40~ 0.92 | ITT: 37.7 vs 32.1,HR=0.93;95% CI:0.77~1.12 pMMR:30.9 vs 29.4,HR=1.02;95% CI:0.83~1.26 dMMR:NR vs NR，HR=0.57;95% CI:0.36~0.91 |
| CAP 04 | ChiCTR2000031932 | Camrelizumab(PD-1)、Rivoceranib(TKI) | II | single-arm | 36 | 44.4(27.9~61.9) | 91.7(77.5~98.2) | 6.2(5.3~11.1) | 21.0(13.4~NR) |
| - | NCT02912572 | Avelumab(PD-L1)/Talazoparib(PARPi) or Avelumab(PD-L1)/Axitinib(TKI) | II | single-arm | 35 | 11.4(3.2~26.7) | - | 3.6(2.4~5.4) | - |
| - | NCT04269200^[27]^ | Durvalumab(PD-L1) ±Olaparib(PARPi) | III | A: chemotherapy+placebo; B：chemotherapy+Durvalumab(PD-L1); C: chemotherapy+Durvalumab(PD-L1)+Olaparib(PARPi) | 718 | - | - | B vs A (10.2 vs 9.6,HR=0.71,95%CI:0.57～0.89,P=0.003)；C vs A (HR=0.55,95%CI:0.43～0.69，P<0.0001） | B vs A(NR vs 25.9,HR=0.77,P=0.120) ,C vs A(NR vs 25.9,HR=0.59,P=0.003) |
| RUBY | NCT03981796^[28]^ | Dostarlimab(PD-1) ,Paclitaxel and Carboplatin | III | A: chemotherapy+placebo; B：chemotherapy+Durvalumab(PD-1); | 494 | - | - | MSI-H/dMMR: A:7.7, B:NE | A:NE;B:NE |
