# Supplementary material for: Advancements in the utilization of immune checkpoint inhibitors for the treatment of gynecological tumors
Source: Front Immunol. 2026 Mar 30;17:1686568. doi: 10.3389/fimmu.2026.1686568 (PMC13071018; doi:10.3389/fimmu.2026.1686568)
Supplement: Supplementary file 1 [file Supplementaryfile1.zip › Supplementary Table 1.DOCX]

Supplementary Table 1. Clinical Trials results for Endometrial Cancer

| Title | Trial number | Treatments | Phase | group | Number(n) | ORR(95%CI) | DCR(95%CI) | mPFS(months, 95%CI) | mOS(months, 95%CI) |
| --- | --- | --- | --- | --- | --- | --- | --- | --- | --- |
| GARNET | NCT02715284 | Dostarlimab(PD-1) | I | single-arm | dMMR:129;pMMR:161 | dMMR:42.3%;  pMMR:15.4% | NR | dMMR:8.1; pMMR:2.7 | dMMR:NR; pMMR:16.9 |
| KEYNOTE-016 | NCT01876511 | Pembrolizumab(PD-1) | II | single-arm | 15 | 53 | 73 | NA | NA |
| KEYNOTE-028 | NCT02054806 | Pembrolizumab(PD-1) | IB | single-arm | 24 | 13(2.8~33.6) | 73 | NA | NA |
| - | NCT01375842 | Atezolizumab(PD-L1) | IA | single-arm | 15 | 13 | 26.6 | 1.4 | 9.6 |
| PHAEDRA/ANZGOG1601 | ACTRN12617000106336 | Durvalumab(PD-L1) | II | single-arm | 71 dMMR:36 pMMR:35 | dMMR：47(32~63);pMMR:3(1~15) | NA | dMMR:5.5,pMMR:1.8 | dMMR:NR;pMMR:11.5 |
| KEYNOTE-158 | NCT02628067 | Pembrolizumab(PD-1） | II | single-arm | 94 | 50(39.5~60.5) | 68 | 13.1(4.3~25.7) | 65.4(29.5~NR) |
